# Capturing atomic wetting dynamics in real time

**DOI:** 10.1038/s41467-025-66416-1

**Published:** 2025-11-28

**Authors:** George T. Tebbutt, Christopher S. Allen, Anna Fabijańska, Barbara M. Maciejewska, Nicole Grobert

**Affiliations:** 1 Department of Materials, University of Oxford, Oxford, United Kingdom; 2 Electron Physical Science Imaging Centre, Diamond Light Source, Harwell Science and Innovation Campus, Didcot, United Kingdom; 3 Łódź University of Technology, Institute of Applied Computer Science, Łódź, Poland

**Keywords:** Nanowires, Carbon nanotubes and fullerenes, Computational nanotechnology, Solid-state chemistry, Surfaces, interfaces and thin films

## Abstract

Atomic-scale wetting governs material formation at the nanoscale but remains poorly understood under confinement, where classical capillarity models fail. The growth of metallic nanowires within multi-wall carbon nanotubes (MWCNTs) exemplifies this challenge, requiring precise control over wetting, nucleation, and vapour-phase condensation. Here we show that nanowire formation proceeds through a two-stage mechanism: curvature-driven nucleation at open tube ends followed by capillary-driven elongation sustained by continuous vapour condensation. Using in situ atomic-resolution transmission electron microscopy (ARTEM) coupled with a deep learning convolutional neural network (CNN) capable of classifying liquid, solid and intermediate Sn_x_O phase transitions, we directly capture the cascade of thermally induced nanowire growth within CNTs. Growth requires a wetting interface (contact angle, *θ* <90°) between liquid Sn_x_O and the nanotube wall—conditions not described by Kelvin or Lucas–Washburn models. These results establish a predictive framework for vapour-phase nanowire encapsulation, linking nanoscale wetting dynamics to the fabrication of advanced nanomaterials.

## Abstract (web summary)

Atomic-resolution microscopy and AI reveal how metallic nanowires grow inside carbon nanotubes through atom-by-atom wetting, advancing understanding of how next-generation materials can be synthesized from the atomic scale up.

## Introduction

Wetting at the atomic-scale is fundamental to achieving the filling of metallic nanowires within the central core of multi-wall carbon nanotubes (MWCNTs), yet the interfacial dynamics that govern their formation remain poorly understood^1–3^, hindering progress in advancing the production of encapsulated metallic nanowire with unique electrical, thermal, and mechanical properties^4–7^.

Under cylindrical confinement, the apparent contact angle, *θ*, determines the axial capillary pressure that drive (or resists) nanowire encapsulation, as described by the Young-Laplace equation^1,8^:

1 $$\Delta P=-\frac{2\,{\gamma }_{{{{\rm{ls}}}}}{{\mathrm{cos\; \theta }}}}{r}$$

where γ_ls_ is the liquid–solid surface tension and *r* are the inner radius of the nanotube. When *θ* < 90°, the pressure is tensile, and a concave meniscus draws liquid into the tube, enabling nanowire growth. When *θ* > 90°, the pressure becomes repulsive, inhibiting nanowire formation.

Unlike wetting on bulk polycrystalline graphite^9,10^, wetting at the nanoscale is governed by the high surface-to-volume ratio amplifies the role of interfacial energy forces, which are essential for nanowire formation in the MWCNT core. While van der Waals adhesion (1/*r*²), curvature-induced Laplace pressure (1/*r*), and line tension at the three-phase contact line (1/*r*) all contribute to influence the apparent contact angle, *θ*^11–13^. These forces, along with curvature-induced capillary pressures, collectively enable metallic vapour to wet and permeate the core of the carbon nanotube (CNT)^1,14–16^. However, at the nanoscale, the wetting dynamics of metallic nanowires within this confined environment are far more complex to assess, probe and evaluate under such extreme conditions of high temperature and vacuum.

Despite decades of reports demonstrating the filling of metals (Fe^17^, SnPb^18^, SnSe^19^), oxides (PbO^20^), salts (CuI^21^, CoI^22^), and even multicomponent perovskites (CsPbI_3_^23^, CsPbBr_3_^24^), within the core of both single- and multi- wall carbon nanotubes, the mechanism that determines whether a metallic vapour forms a nanowire inside the nanotube core **–** or is excluded **–** remains empirical^4,5^.

For vapour-phase filling of nanocapillaries in MWCNTs, wetting does not proceed via continuous inflow of liquid but rather through a cascade of transient steps: vapour adsorption, nucleation, meniscus formation, and subsequent nanowire growth. Classical models like the Kelvin^25^ and capillary filling (Young-Laplace^2,26^) theories describe the idealised equilibrium processes, but neglect the nucleation kinetics, adsorption threshold, and the role of local vapour saturation that governs which nanowires can be grown, how fast, and to what extent, i.e. filling length.

**Fig. 1:**
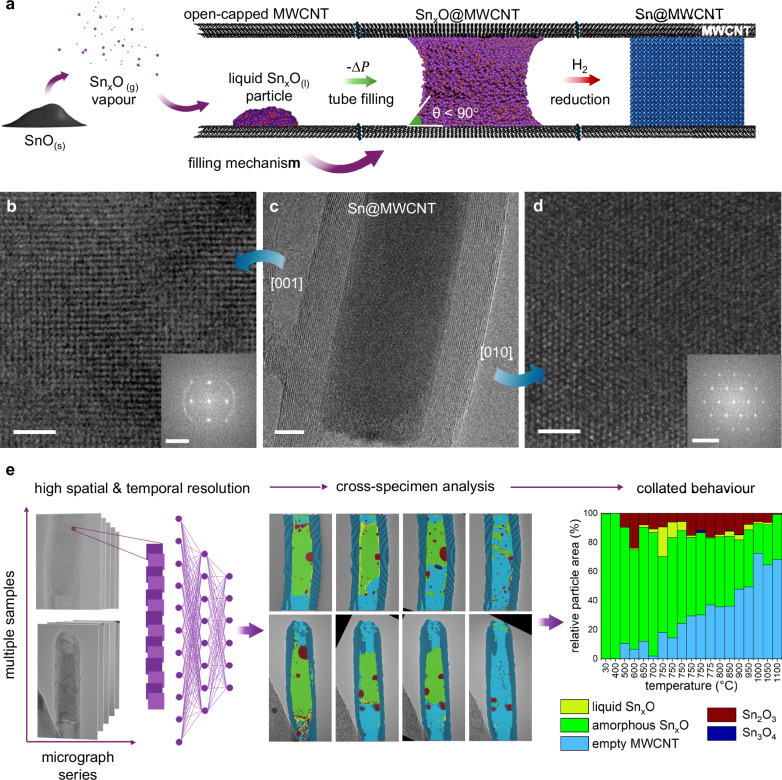
Encapsulation of Sn_x_O and CNN-assisted analysis of the dynamic encapsulation process leading to β-Sn encapsulation within MWCNTs. **a** Schematic illustrating the sublimation of  SnO and its disproportionation into Sn_x_O vapour. This vapour enters the open end of a MWCNT, enabling the encapsulation of Sn_x_O nanowires (Sn_x_O@MWCNT). Subsequent reduction in a hydrogen atmosphere converts these nanowires which are transformed into fully encapsulated, single-crystalline metallic β-Sn nanowires (Sn@MWCNT). **b** High-resolution ARTEM micrograph of a Sn nanowire inside an MWCNT, viewed down the cubic [001] zone axis. Inset: corresponding Fast Fourier Transform (FFT) confirming the [001] orientation. **c** ARTEM micrograph of a typical single-crystal encapsulated β-Sn nanowire, showing the orthogonal relationship between the [001] and [010] nanowire orientation. **d** High-resolution ARTEM micrograph of a single-crystal β-Sn nanowire viewed down the tetragonal [010] zone axis. Inset: FFT confirming the tetragonal symmetry. **e** Schematic framework illustrating the application of convolutional neural networks (CNNs) for ARTEM micrograph analysis. Micrographs are segmented into 64 × 64 pixel patches, which are processed by the CNN to classify atomic-scale texture feature across the datasets. This automated labelling enables cross-specimen analysis by classifying features of the nanowire growth mechanism by class type. By applying this analysis across hundreds of frames, the CNN facilitates statistically robust insight into nanowire growth and identifying global trends in the encapsulation mechanism. Scale bars: **b** 3 nm and 3.5 nm^−1^, **c** 5 nm, **d** 5 nm and 7 nm^−1^.

Herein, we address this mechanistic gap by studying Sn-filled MWCNTs as a model system, using in situ atomic-resolution transmission electron microscopy (ARTEM) in conjunction with advanced data processing and image analysis. We synthesised Sn-filled MWCNTs by first introducing SnO vapour, then reducing the encapsulated Sn_x_O nanowire in a hydrogen atmosphere to yield β-phase single-crystalline Sn, as schematically shown in Fig. 1a (see Methods for details). Figure 1b shows the corresponding ARTEM micrographs illustrating the crystallinity and orientation of the confined Sn nanowire.

Supplementary Figures  1 and 2 show β-Sn nanowire imaged along two orthogonal zone axes:one projection appears pseudo-cubic due to its viewing direction, while the other clearly reveals the characteristic tetragonal symmetry of the β-Sn structure. These are presented alongside lattice parameter analysis and nanowire orientation analysis in the Supplementary Section 1. Detailed characterisation of Sn_x_O encapsulation is provided in the Supplementary Section 2.

## Results

### Direct in situ ARTEM

To capture the thermally induced solid–liquid–vapour transitions relevant to nanowire formation, we imaged the dynamic phase evolution of Sn_x_O in real-time under high-vacuum, high-temperature conditions within a transmission electron microscope (TEM). While these observations reflect the reverse sequence of nanowire growth, they reveal direct insight into the wetting interface behaviour, interfacial condensation, and the liquid-phase dynamics that govern confined nanowire formation within MWCNTs. Pre-encapsulated Sn_x_O-filled MWCNTs were deposited on a DENSsolutions silicon nitride heating chip, which was progressively heated from ambient temperature to 1100 °C, while simultaneously acquiring ARTEM micrographs at each set temperature.

Modern ultra-fast in situ ARTEM detectors can record structural changes such as movements at the atomic level at a rate of over 1000 frames per second, yielding processing-intensive datasets^27^, which are impractical to analyse manually. To handle such large and multi-dimensional datasets, we developed a deep learning CNN designed to track these atomic movements across hundreds of ARTEM micrographs. Collectively, these atomic movements recorded frame-by-frame reveal the atomic-scale evolution from initial nucleation to nanowire formation.

The CNN processes each micrograph using a local-context, patch-based approach. A 64 × 64 pixel window that rasters across the image, classifying each central pixel based on its surrounding atomic texture. The model was pre-trained on approximately 1,000,000 manually annotated patches from ARTEM images of Sn_x_O-filled MWCNTs, each representing characteristic atomic textures for each pixel class. By applying this analysis across multiple frames and samples, the CNN captures statistically robust vapour-phase dynamics and can resolve global trends in the nanowire growth mechanism.

Figure 1c presents a schematic of the CNN workflow designed to extract statistical insights from the ARTEM micrographs. Details on the CNN architecture and experimental workflow are provided in the Suppl. Section 3 and Methods section (see Deep learning CNN).

**Fig. 2:**
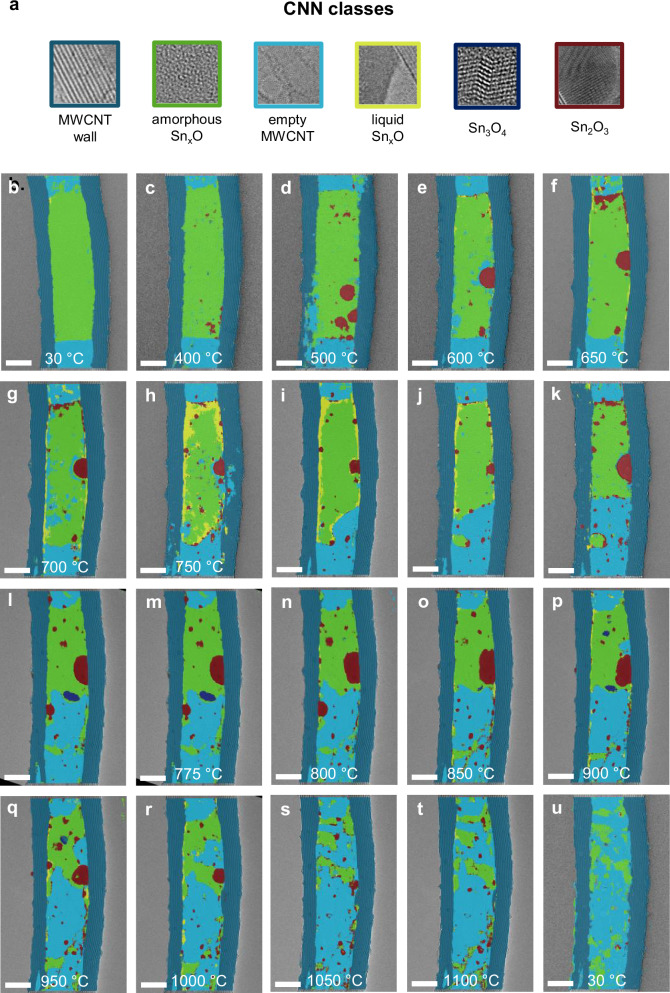
Atomic textured-based nanowire tracking using CNN convolution neural network. **a** Example of the seven textural classes used to train the CNN model: the MWCNT wall (blue), empty MWCNT tube (light blue), encapsulated solid amorphous Sn_x_O (green), the liquid-phase Sn_x_O (yellow), and intermediate oxides (Sn_3_O_4_, dark blue and metallic Sn_2_O_3_, dark red), formed during Sn_x_O disproportionation, and unlabelled background. **b**–**u** CNN-labelled micrographs showing the morphological evolution and vaporisation of a encapsulated Sn_x_O nanowire as a function of increasing temperature: 30 °C (static), **c** 400 °C, **d** 500 °C, **e** 600 °C, **f** 650 °C, **g** 700 °C, **h**–**l** 750 °C (25 s between each micrograph), **m** 775 °C, **n** 800 °C, **o** 850 °C, **p** 900 °C, **q** 950 °C, **r** 1000 °C, **s** 1050 °C, **t** 1100 °C, **u** 30 °C (upon cooling). Scale bar: **b**–**u** 10 nm.

Figure. 2 presents a CNN-labelled in situ heating series, where the model distinguishes key pixel classes involved in the encapsulation process: the MWCNT wall (blue), the solid phase of the encapsulated amorphous Sn_x_O nanowire (green), the liquid-phase Sn_x_O (yellow), intermediate crystalline oxides (Sn_3_O_4_, dark blue and Sn_2_O_3_, dark red) formed by disproportionation, and the empty MWCNT core (light blue).

The corresponding unprocessed ARTEM micrographs are provided in the Supplementary Section Fig. 10. The processing of a second in situ ARTEM dataset presented in ﻿Supplementary Fig. 11, with corresponding CNN processed micrographs in ﻿Supplementary Fig. 12.

Applied frame-by-frame to the in situ ARTEM series (Fig. 2b–u), the CNN tracks the evolution of the nanowire through three distinct thermally induced phase states: green amorphous solid, a yellow liquid phase along the CNT wall, and light blue representing empty nanotube core as liquid Sn_x_O enters the vapour phase leaving it vacant. The sequence reveals a series of non-equilibrium, interface-driven events following melting, wetting, and vapourisation. Upon heating, the encapsulated nanowire transitions from solid to liquid, accompanied by wetting of the nanotube wall, followed by gradual vaporisation, evident as continuous shrinkage of the nanowire.

The initial amorphous morphology of the Sn_x_O nanowire (green) is shown in Fig. 2b. Initial heating to 400 °C (Fig. 2c), reveals the onset of re-disproportionation of Sn_x_O, marked by the formation of crystalline metallic oxides (dark red and dark blue) domains which are seen as dark particles within the bright-field (BF) micrograph contrast (Supplementary Figures 1 and 2). Lattice-resolved identification and the evolution of transient triclinic *P*_*1*_ Sn_2_O_3_^28^ (﻿Supplementary Figs. 13 and 14) and orthorhombic *Pbcm* Sn_3_O_4_^29^ (﻿Supplementary Fig. 15) intermediate oxides are discussed in detail in ﻿Supplementary Section 5. Heating the Sn_x_O nanowire to 750 °C produces a curved meniscus against the CNT wall (Fig. 2g–j), indicative of a condensed liquid phase (yellow). As vapourisation ensues, the liquid meniscus retracts frame-by-frame, accompanied by a decrease in the yellow-labelled area as the vapour exits the nanotube. Continued heating (Fig. 2k–u) causes the Sn_x_O nanowire to vaporise within the nanotube core.

Our in situ observations reveal that nanowire growth within the MWCNT proceeds through a confined, transient liquid phase, stabilised by the nanoscale confinement effects consistent with Kelvin theory. ADF-STEM images (﻿Supplementary Fig. 16) reveal Sn_x_O nanowires consistently positioned just inside the open-ended nanotube. This spatial localisation of Sn_x_O nanowires supports a Kelvin-driven condensation mechanism: as SnₓO vapour enters the confined nanotube, the reduced equilibrium vapour pressure favours localised condensation into a liquid droplet, which subsequently elongates inward under capillary forces and solidifies into a nanowire. The liquid–solid interface against the nanotube wall dictates the compatibility for a nanowire to grow within the graphitic core. Effective wetting and adhesion at the interface are critical for initiating and sustaining the growth of metallic nanowires. These observations present a multi-stage filling mechanism: vapour molecules first adsorb at the open MWCNT ends, nucleate localised liquid droplets, and subsequently condense to sustain the axial advance of a molten nanowire driven by capillary forces.

Unlike an equilibrium pathway governed purely by surface tension and the geometry of the nanotube, tube filling is mediated by a cascade of transient interfacial interactions, namely vapour adsorption, nucleation, wetting, and vapour-phase condensation, all under nanoscale confinement. While the Kelvin and Laplace theorems provide the baseline description of condensation and capillary rise in closed cylindrical pores, they fail to capture the kinetics or boundary conditions relevant to open ends of the MWCNT. In this context, MWCNTs behave more like quasi-one-dimensional capillaries, where condensation does not occur uniformly throughout the volume but instead proceeds directionally from the open ends of MWCNTs.

In contrast to assumptions from classical capillary condensation theory, which predicts the formation of a continuous adsorbed wetting film prior to condensation, we observe no such film—either by direct imaging or Electron Energy Loss Spectroscopy (EELS; see ﻿Supplementary Section 7). Instead, condensation proceeds via discrete nucleation events, indicating the presence of kinetic barriers that must be overcome to initiate nanowire growth.

### Wetting: contact angle and its role in capillary filling

**Fig. 3:**
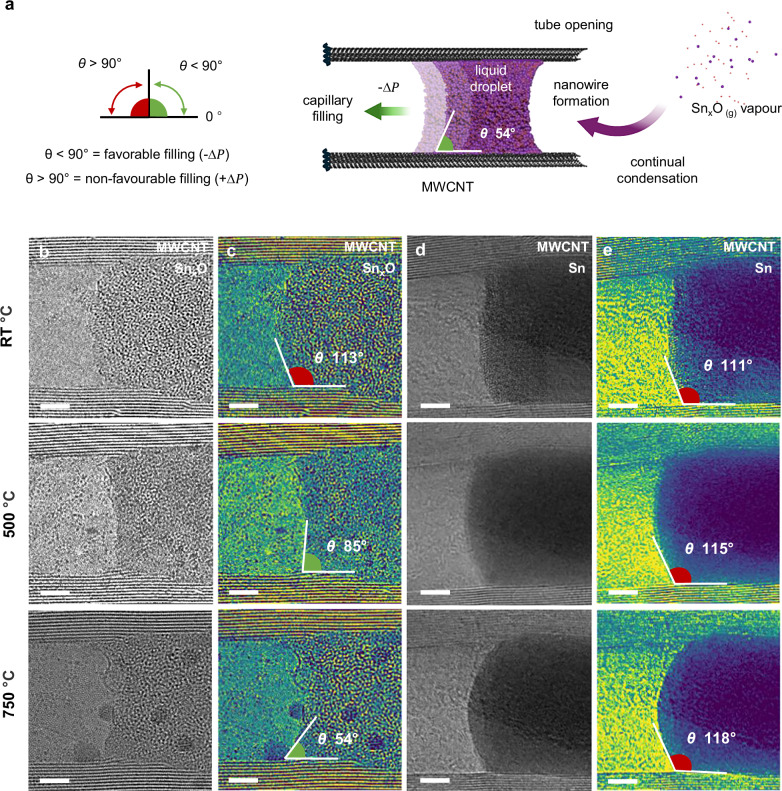
Evaluating wettability towards nanowire growth. **a** Schematic illustration of encapsulates liquid-phase Sn_x_O inside a MWCNTs, demonstrating how tube wetting governs capillary-driven filling. When the contact angle, *θ*, is <90°, a concave meniscus forms, generating a tensile capillary pressure (−ΔP) that stabilises the nanowire and promote nanowire growth within the nanotube core. In contrast, *θ *> 90° indicates poor tube wetting and yields a repulsive capillary pressure (+ΔP), inhibiting nanowire formation and growth. **b**, **c** ARTEM and contrast-adjusted micrographs for Sn_x_O nanowire at 23 °C, 500 °C, 750 °C, showing the progressive reduction in contact angle, *θ*, with temperature, indicative of wetting behaviour. **d**, **e** ARTEM and contrast-adjusted Sn nanowire over 23 °C, 500 °C, 750 °C, showing consistently obtuse contact angles indicative of non-wetting behaviour. Scale bar: **a**–**d** 3 nm.

To examine how wetting evolves with temperature, we measured the apparent contact angle, *θ*, of Sn_x_O and Sn nanowires at the MWCNT interface. As illustrated in Fig. 3a, in accordance with Young-Laplace capillary theory, the Sn_x_O nanowire exhibits favourable wetting (*θ* < 90°), enabling capillary-driven filling, whereas Sn remains non-wetting (*θ* > 90°), inhibiting nanowire formation.

Due to the cylindrical confinement of the MWCNT, the droplets adopt a spherical geometry, rendering contact angle, *θ,* largely independent of tube diameter. Instead, the contact angle, *θ*, is governed by the balance between temperature-dependent van der Waals adhesion and liquid surface tension.

In situ ARTEM imaging (Fig. 3b, c) shows a contact angle, *θ*, for Sn_x_O decreases from 113° at 400 °C to 54° at 750 °C, exhibiting a wetting transition. This is attributed to the reduced surface tension and strong adhesion of Sn_x_O to the CNT wall, which promotes meniscus spreading and sustains nanowire growth along the graphitic interface.

In contrast, encapsulated Sn nanowire remains non-wetting, with the contact angle, *θ*, increasing from 111° at 23 °C to 118° at 750 °C (Fig. 3d, e), reflecting stronger cohesive forces within the  Sn melt than adhesive interactions with the CNT wall.

Comparative contact angle measurements (Supplementary Fig. 19) show that Sn_x_O becomes wetting above 600 °C (beyond its melting point) whereas Sn remains non-wetting, owing to the oxygen-enhanced polarisability and stronger adhesion of Sn_x_O at the CNT interface. This comparison indicates that confinement alone does not enhance wetting; rather, capillary-driven encapsulation depends critically on the chemical nature of the nanowire and its interfacial compatibility with the graphitic wall. In this context, the MWCNT thus act as nanoscale test tubes, enabling direct, high-resolution observation of the interfacial behaviour of Sn across an extended temperature range (30–1000 °C).

For comparison, the nanoscale wetting of encapsulated Sn droplets is presented alongside macroscale sessile-drop measurements^9^ in Supplementary Fig. 20. Both exhibit similar non-wetting average contact angles (*θ*  =  120–125° on bulk graphite between 400–600 °C) demonstrating that Sn remains non-wetting across scales. However, at the nanoscale, the measured contact angles, *θ*, show significantly larger error bars, reflecting increased variability in local wetting behaviour.

At these dimensions, the droplet radius becomes comparable to structural inhomogeneities along the CNT wall, such as point defects, step edges, vacancies, or amorphous regions, which perturb the interfacial energy landscape at the three-phase contact line. These atomic-scale features modify both line tension and local adhesion, causing site-specific deviations in contact angle, *θ*, even under globally uniform conditions. For nanofabrication, this has important consequences: although the average wetting behaviour remains unchanged, atomic defects can locally alter wetting, influencing where capillary-driven ingression occurs and how nanowires nucleate, elongate, or terminate. As a result, even isolated atomic-scale defects can impact growth morphology and reproducibility, highlighting the need for precise surface control when fabricating confined metallic structures.

### Growth mechanism of tube filling

To complement our in situ observations of interfacial wetting and liquid-phase behaviour, we analysed the statistical evolution of encapsulated Sn_x_O nanowires as a function of nanotube diameter and vapour annealing times. By measuring the nanowire length and filled diameter over vapour annealing time, we identified a transition from an early mechanism dominated by capillary forces to a later regime dominated vapour-phase condensation.

**Fig. 4:**
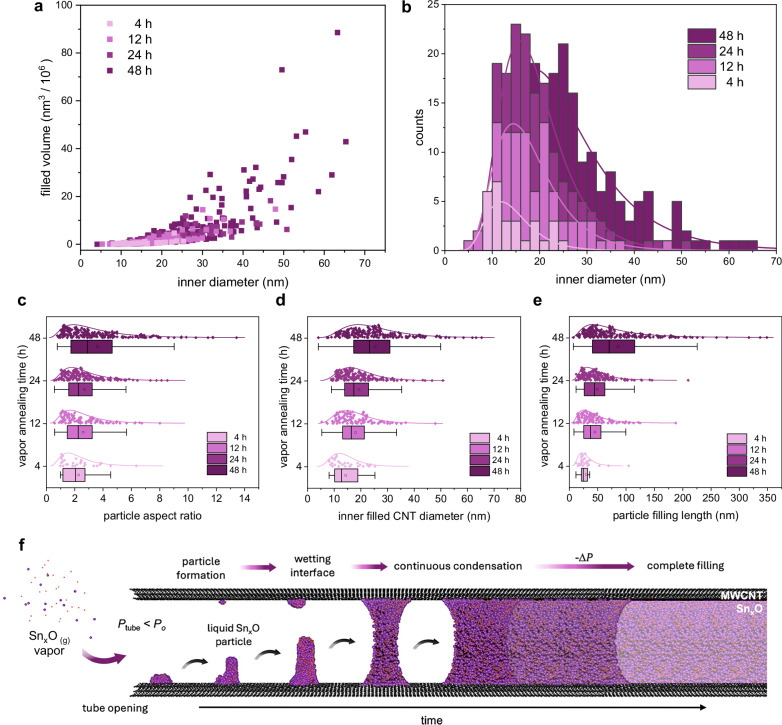
Mechanism of nanowire growth *via* confined condensation and wetting. **a** Scatter plot showing the relationship between filled volume and inner diameter of Sn_x_O-filled MWCNTs after exposure to Sn_x_O vapour for 4 h (light pink), 12 h (pink), 24 h (purple), and 48 h (dark purple) at 850 °C. Longer annealing times lead to increased filling, particularly in larger-diameter tubes. The colour gradient from light to dark purple represents progression from early to extend vapour annealing times. **b** Corresponding histogram of filled MWCNT diameters at each annealing, illustrating the progressive filling of large diameter over greater annealing time—consistent with increasing vapour saturation and diameter-selective nucleation threshold. Box plots of (**c**) filled diameter, (**d**) nanowire length, and (**e**) aspect ratio (length normalised by diameter) as a function of annealing time, illustrating the statistical evolution of nanowire distribution over time. **f** Schematic illustration of the two-stage growth mechanism: Sn_x_O vapour condenses near the open tube end to nucleate a liquid droplet, whereby the internal pressure is less within the tube (*P*_tube_) is greater than the external vapour pressure (*P*_o_). The resulting condensation droplet proceeds to growth and forms a wetting interface against the MWCNT wall (*θ* < 90°). Sustained condensation at the liquid-vapour interface drives capillary nanowire growth along the MWCNT core, ultimately forming a continuous Sn_x_O nanowire (lighter shaded Sn_x_O).

Figure 4a shows a scatter plot of the filled volume and nanotube diameter for each annealing time: 4 h (light pink), 12 h (pink), 24 h (purple), and 48 h (dark purple). Early filling is dominated by smaller diameter MWCNTs (6–18 nm), while larger diameters (< 30 nm) increasingly contribute to the total filled volume at longer annealing times.

Figure 4b displays histograms of filled tube diameters for each annealing point, showing that smaller-diameter tubes dominate at early annealing times, while progressive filling of larger-diameter tubes occurs at extended annealing durations. The initial diameter distribution of unfilled MWCNTs (0 h) is shown in Suppl Fig. 21.

To quantify this behaviour, we applied the Lucas-Washburn model^30^, which predicts that the length, *L*, of a liquid meniscus advancing in a cylindrical tube that scales with the square root of time ($\sqrt{t}$), due to the balance of capillary forces and opposing viscous drag along the nanotube walls (see Supplementary. Section 10).

For cylindrical geometries, the filled nanowire volume, *V*, scales with nanotube diameter, *d*, as a power law:

2 $$V={{ad}}^{2.5}$$

The prefactor, α, governs condensation efficiency as a function of temperature, vapour pressure, and wetting dynamics. This prefactor increases with annealing time, indicating enhanced filling efficiency in larger-diameter MWCNTs. These larger tubes exhibit higher nucleation thresholds and thus require greater local saturation vapour pressures to initiate nanowire growth. Conversely, smaller MWCNTs (< 10 nm) nucleate earlier due to the higher curvature of the nanotube diameter, in agreement with Kelvin condensation theory.

Post-nucleation, nanowire growth follows the power-law model described in Eq. 2 and is driven by capillary forces rather than vapour flux limitations. Each annealing time series shows strong agreement with this model (see Supplementary Fig. 22), supporting the mechanistic observations that, once nucleation occurs, capillary forces govern elongation and vapour flux is no longer rate-limiting.

Small-diameter MWCNTs fill preferentially at earlier annealing times, depleting the local vapour and establishing a saturation gradient. As these tubes become saturated or partially filled, viscous resistance to nanowire growth increases, local pressure builds near larger MWCNTs. This gradual pressure increase eventually surpasses the higher nucleation thresholds of larger-diameter nanotubes, triggering nucleation and enabling subsequent nanowire growth.

In larger-diameter MWCNTs (> 25 nm), delayed nucleation gives way to rapid nanowire growth, proceeding at a rate greater than predicted by Lucas-Washburn theory due to the fast kinetics of vapour condensation. Growth continues until viscous drag equilibrates with vapour pressure, gradually decelerating the capillary-driven growth of the nanowire. Once the nucleation barrier is overcome, nanowire growth is thermodynamically driven by capillary forces and sustained by continuous condensation at the liquid-solid interface.

Nanowire growth rates derived from the median box-plot distributions in Fig. 4c–e indicate a filling length of 2.30 ± 0.64 nm h^−1^, a diameter filling rate of 0.78 ± 0.27 nm h^−1^, and an aspect ratio gain of 0.11 ± 0.04 h^−1^ with increasing vapour exposure (see Supplementary. Table 8 for summary of statistics).

Figure 4f illustrates a two-stage mechanism for SnₓO vapour-phase encapsulation in MWCNTs. In the first stage, vapour condenses *via* curvature-driven nucleation to form a wetting liquid interface. In the second stage, nanowire growth proceeds through capillary-driven elongation, sustained by continued condensation at the curved liquid–solid interface.

Classical Kelvin and Lucas–Washburn models, which assume equilibrium condensation and spontaneous capillary flow, fail to capture the dynamic, non-equilibrium nature of vapour-phase encapsulation in MWCNTs. Our in situ observations reveal that the process is governed by discrete nucleation events: vapour molecules first adsorb onto the inner walls of the MWCNT, subsequently form stable liquid nuclei that must overcome the nucleation energy barriers—making nucleation is the rate-limiting step. High curvature and strong surface interactions reduce these barriers, enabling earlier filling in smaller-diameter MWCNTs, consistent with Kelvin theory. Although elevated vapour saturation accelerates nucleation, encapsulation remains dominated by time-dependent interfacial kinetics rather than equilibrium thermodynamics. Once a wetting interface (*θ* < 90°) forms, capillary forces drive nanowire growth.

Our findings provide direct mechanistic insight into the interfacial principles governing vapour-phase encapsulation of metallic wires. First, precursor chemistry determines encapsulation: only species that exhibit favourable wetting interactions with the graphitic MWCNT wall, such as Sn_x_O, can condense, nucleate, and grow within the nanotube core. Second, wetting interactions at the MWCNT wall control both the onset and directionality of growth, with temperature-dependent contact angle, *θ*, transitions enabling nanowire elongation. Third, diameter-dependent filling reveals that smaller MWCNTs nucleate earlier due to higher curvature and lower condensation thresholds, while larger tubes, once nucleated, grow more rapidly due to increased cross-sectional area, higher vapour flux, and reduced viscous resistance. Finally, nanowire growth following nucleation is not limited by vapour flux at the meniscus, but is sustained by local vapour saturation, which continuously drives condensation at the advancing liquid–solid interface.

In situ ARTEM combined with CNN-based micrograph analysis reveals a two-stage mechanism for nanowire encapsulation in MWCNTs: curvature-induced nucleation at open nanotube ends, followed by capillary-driven nanowire growth sustained by vapour-phase condensation. Central to this process is nanowetting:the formation of a temperature-dependent, energetically favourable liquid interface that governs whether a precursor adheres to the nanotube wall and forms a directional meniscus. For favourable wetting precursors, wetting is temperature-dependent; with Sn_x_O exhibiting a transition to a wetting interaction at 600 °C that initiates encapsulation.

Our findings demonstrate that the filling process is governed by the non-equilibrium nucleation kinetics, vapour flux, and interfacial condensation, which are in contrast to the equilibrium-based Kelvin and Lucas-Washburn models. The progression of encapsulation reflects a confinement-governed sequence, where smaller-diameter nanotubes initiate growth earlier due to lower nucleation barriers, while larger diameters exhibit delayed but more complete filling. The resulting nanowire volume scales super-linearly with tube diameter (*V* ∝ *d*^2.5^), underscoring the influence of vapour flux and nucleation kinetics – factors not accounted for by classical models such as Kelvin or Lucas–Washburn. Nanowire growth rates and lengths are dictated not by equilibrium thermodynamics, but by vapour pressure, temperature, and viscous drag.

By establishing a direct link between atomic-scale wetting, dynamic condensation, and capillary-driven growth, our framework enables predictive control over vapour-phase nanowire synthesis. These insights provide a general route for fabrication of confined nanowire architectures by tuning interfacial chemistry, offering broad utility in the bottom-up fabrication of nanoscale devices – from energy storage to quantum transport applications.

## Methods

### Fabrication of MWCNTs

MWCNTs were synthesised via Aerosol-Assisted Chemical Vapour Deposition (AACVD) using a piezo-driven aerosol generator to deliver the precursor solution^31, 32^. The precursor consisted of 5 wt% ferrocene (C_10_H_10_Fe, 99%, Sigma Aldrich, sublimed at 100 °C) dissolved in 95 wt% toluene (C_6_H_5_CH_3_, 98%, Thermo Fisher Scientific). The generated aerosol was carried by argon (Pureshield, BOC Ltd, UK) at a flow rate of 1500 sccm into a horizontal quartz tube reactor (60 cm length, 22 mm inner diameter) positioned within a tube furnace maintained at 850 °C. MWCNTs were collected from the central hot zone of the reactor after a 3 h growth period. Prior to growth, the system was purged with argon to remove residual atmospheric gases and ensure an inert environment.

### MWCNT thermochemical oxidation

MWCNTs (250 mg) were refluxed in nitric acid (150 mL, HNO_3_, 70%, analytical grade, Sigma Aldrich) within a three-neck round-bottom flask. The mixture was heated to 112 °C and stirred for 18 h using a heating mantle. After cooling naturally, the oxidised MWCNTs were separated from the reagent mixture by centrifugation (diameter 18.5 cm) at 4600 rpm (4373 × *g*) for 5 min, followed by sequential solvent washes. The acidic supernatant was discarded, and the MWCNT sediment was redispersed in an ethanol-de-ionised water mixture (30 mL, 1:1 v/v, ≥99.8%, Sigma Aldrich) and centrifuged at 4600 rpm (4373 × *g*) for 10 min at 20 °C. The supernatant was discarded, and this step was repeated in 2-propanol (30 mL, ACS reagent grade, ≥99.8%). The resulting sediment was collected and dried in vacuo at 333 K overnight, yielding 180 mg of open-capped oxidised MWCNTs.

### MWCNT sponge synthesis

Oxidised MWCNTs were dispersed in deionised water (1 mg mL^−1^) and sonicated (60 Hz) for 1 h at 323 K. The solution was then submerged in liquid nitrogen for 5 min and subsequently freeze-dried at 223 K for several days until the vapour pressure plateaued.

### Encapsulation of Sn_x_O@MWCNT

Sublimation SnO (tin(II) oxide) precursor powder (100 mg, 400 mesh, 99.9%, metal basis, Thermo Scientific) was placed inside a modified quartz tube ampoule (O.D. 16 mm, I.D. 14 mm), with the MWCNT sponge (10 mg) positioned at the opposite end. The ampoule was sealed under partial vacuum (10⁻^4^ Torr), with the SnO precursor end placed in the central zone of a 60 cm long furnace. Glass wool insulated each end of the furnace. The furnace was heated to 1043 K at 10 K min^−^¹ for 4–96 h and then allowed to cool naturally to room temperature.

### Reduction of Sn_x_O@MWCNT

The Sn_x_O@MWCNT sample was exposed to a reductive hydrogen-argon atmosphere (50 sccm Ar, 200 sccm H_2_) in a quartz tube (125 cm) within a 60 cm furnace at 1275 K, heated at 10 K min^−^^1^ for 24 h, to produce metallic Sn-filled MWCNTs (Sn@MWCNT). The system was allowed to cool naturally. Sn@MWCNT samples (50 mg) were refluxed in HCl (6 M, 50 mL, HCl, 37%, 12 M, Fischer Scientific) for 2 h to remove external Sn deposits.

### Static TEM imaging and spectroscopy

The morphology, elemental composition, and crystallinity of MWCNTs, Sn_x_O@MWCNT, and Sn@MWCNT were investigated using a JEOL ARM200F microscope (Cs-corrected probe) operated at 80 kV in both TEM and STEM imaging modes. The ARM200F was equipped with a Gatan Quantum Enhanced Resolution system for imaging, a JEOL annular dark-field (ADF) detector, fast dual Electron Energy Loss Spectroscopy (EELS) capabilities, and a 100 mm² Oxford Instruments Centurion energy-dispersive spectroscopy (EDS) detector. EDS spectra were acquired across the filled MWCNTs, with analysis performed using commercially available Aztec software (version 6.1). The Gatan Image Filter (GIF) detector was segmented to simultaneously capture the zero-loss peak, carbon K-edge at 284 eV, and the oxygen K-edge at 534 eV, with an energy dispersion of 0.25 eV per channel and an energy resolution at the zero-loss peak of 0.75 eV. EELS spectroscopy was conducted using a line scan profile along the CNTs, with all spectra aligned to the zero-loss peak. EELS data analysis was carried out using commercially available Gatan Digital Micrograph software (version 3.5). Atomic-resolution TEM (ARTEM) was further conducted using a JEOL double Cs-corrected ARM300CF microscope, operated at 80 kV in TEM mode and equipped with a Gatan OneView 4 K camera. For both ARTEM imaging and EELS spectroscopy, sample drift was compensated for using the drift correction feature in the Digital Micrograph software. TEM samples were prepared by drop casting a sonicated solution (1 mg mL⁻¹, 1 h, 45 °C) in ethanol onto Cu TEM grids (400 mesh, lacey carbon, Agar Scientific), followed by drying in vacuo at 60 °C for 6 h. All ARTEM micrographs were calibrated to the reliable MWCNT wall d-spacing of 0.335 nm.

### In situ TEM sample preparation and heating

A dispersed solution of Sn_x_O@MWCNT or Sn@MWCNT in ethanol was directly drop-cast onto a heating chip with a silicon-nitride (Si_3_N_4_) support. The chip was then heated to 60 °C under vacuum to remove residual solvent. The Si_3_N_4_ support layer was modified using gallium focused ion beam (Ga-FIB) milling to drill a series of 5 µm holes, facilitating high-resolution imaging in vacuum conditions. Samples were imaged using a JEOL ARM300CF double Cs-corrected transmission electron microscope, operated at 80 kV, with micrographs captured on a Gatan OneView 4 K camera. Prior to heating the sample to 1100 °C, a preheating step at 300 °C for 1 h was conducted to remove adsorbed surface residues and evacuate air within the inner core of the nanotubes. No morphological changes were observed during this preheating process. The sample was subsequently heated in increments of 10–100 °C and held for imaging. Samples were prepared direct onto microelectrochemical chips (DENSolution V2 chips) via drop casting a sonicated solution (0.2 mg mL⁻¹, 1 h, 45 °C) in ethanol. The DENS Solutions chips are calibrated using Raman spectroscopy and are widely accepted in the community for their temperature accuracy and stability.

### Deep learning CNN

A convolutional neural network (CNN) was trained to classify pixels using 64 × 64 pixel patches extracted from TEM micrographs, with cropped ARTEM micrograph resolutions ranging from *c.a*. 1500 × 2500 to 2500 × 2500 pixels. Manual annotations identified regions such as MWCNT walls, liquid Sn oxide, amorphous Sn oxide, intermediate oxide crystal structures, and the empty nanotube core. Depending on the experiment, the model was trained using between 19 and 22 labelled micrographs, drawn from two separate experimental datasets (comprising over 240 micrographs between both data sets), which were divided into smaller patches, yielding *c.a*. 1,000,000 individual training patches, including data augmentation (rotation, brightness, noise), to achieve an accuracy of >97%. Each patch was labelled based on its central pixel, corresponding to the region type. The model analysed these patches through texture-based recognition, detecting patterns in the pixel intensity variations surrounding each central pixel. To ensure consistency across datasets, each TEM micrograph was spatially standardised by normalising pixel density (for ARTEM micrographs taken at high magnification) such that key atomic features (e.g., MWCNT walls) occupied a consistent number of pixels. Bicubic interpolation was used to resample the images, enabling the CNN to learn and apply features across micrographs acquired at different magnifications or sampling rates. This approach enabled the model to differentiate between regions of atomic contrast, allowing for classification of textural features in unseen micrographs. Further details and a full description of micrograph processing, micrograph labelling, and the design and application of the CNN model architecture are provided Supplementary Section 3.

### Crystal structure visualisations

Structural simulations were performed using commercially available CrystalMaker V10 software to model and visualise atomic structures and crystallographic faces.

## Supplementary information

Supplementary Information

Transparent Peer Review file

## Source data

Source Data

## Data Availability

The experimental and analytical data generated in this study are provided within the paper and its Supplementary Information. Source data for all figures are available with this paper in the accompanying Source Data file. The full training and testing datasets used for CNN development are large and protected under intellectual property review. These data are available for academic use upon request to the corresponding author. Source data are provided with this paper.

## Code availability

The custom CNN segmentation code generated in this study has been deposited in the Zenodo repository under 10.5281/zenodo.17438548. This repository provides a lightweight, demonstration-only version of the segmentation framework, containing one training and one testing image, pretrained weights, and minimal data to reproduce the workflow. The demonstration code reproduces the analysis logic and computational pipeline presented in this study but does not reproduce the exact results, as the full training and testing datasets are not included. The complete framework and datasets are protected under intellectual property review and are available for academic collaboration upon request from the corresponding author.
