# Supplementary material for: Capturing atomic wetting dynamics in real time
Source: Nat Commun. 2025 Nov 28;16:11350. doi: 10.1038/s41467-025-66416-1 (PMC12727699; doi:10.1038/s41467-025-66416-1)
Supplement: Supplementary file 1 — Supplementary Information [file 41467_2025_66416_MOESM1_ESM.pdf]

Supplementary Information for:
‘Capturing atomic wetting dynamics in real time’

George T. Tebbutt[1], Christopher S. Allen[1,2], Anna Fabijańska[3], Barbara M. Maciejewska[1], Nicole Grobert[1]*

[1]*Department of Materials, University of Oxford, Oxford, OX1 3PH, United Kingdom.*

[2]*Electron Physical Science Imaging Centre, Diamond Light Source, Harwell Science and Innovation Campus, Didcot, OX11 ODE, United Kingdom.*

[3]*Lodz University of Technology, Institute of Applied Computer Science, 18 Stefanowskiego Str., 90-537 Lodz, Poland.*

*nicole.grobert@materials.ox.ac.uk

Supplementary Contents:

1. Crystallisation and orientation analysis of encapsulated Sn nanowires 2

2. Disproportionation behaviour of SnO 6

3. Convolution Neural Network (CNN) micrograph processing: 10

4. *In situ* ARTEM and CNN processed micrographs: 14

5. *In situ* formation of Sn_3O_4 and Sn_2O_3: 18

6. Angular dark-field - scanning transmission electron microscopy (ADF-STEM) 22

7. Electron Energy Loss (EELS) Analysis 23

8. Assessing wettability via contact angle analysis 25

9. Filling distribution of Sn_xO nanowires growth over time 27

10. Application of the Lucas–Washburn Model to Nanotube Filling Dynamics: 28

11. Box plot statistic of growth dynamics 33

12. References: 34

1. Crystallisation and orientation analysis of encapsulated Sn nanowires

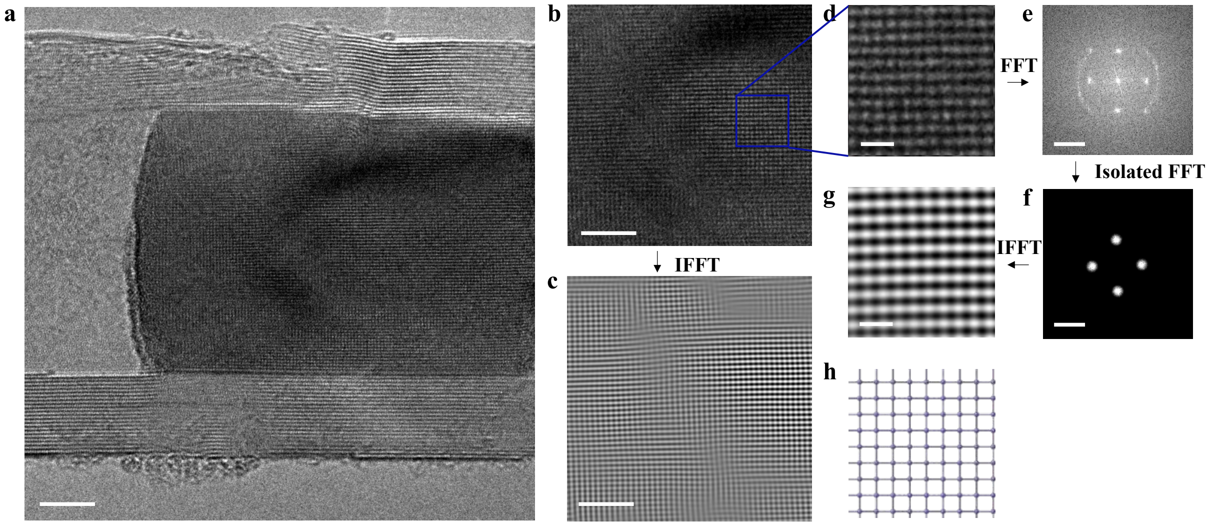

Suppl. Fig. 1: Tetragonal β-phase of a Sn nanowire encapsulated within a MWCNT imaged along the [001] zone axis. a, *bright-field atomic-resolution TEM (BF-ARTEM) micrograph showing the single-crystal nature of the encapsulated Sn nanowire.* ***b,*** *cropped region of the ARTEM micrograph (blue box inset), selected for detailed analysis.* ***c,*** *inverse Fast Fourier transform (iFFT) of the cropped ARTEM micrograph, highlighting the positions of Sn atoms across the nanowire.* ***d,*** *magnified ARTEM micrograph displaying the tetragonal crystal face of β-Sn.* ***e,*** *Fast Fourier transform (FFT) of the magnified ARTEM micrograph.* ***f,*** *isolated mask of the FFT spatial frequencies, used to extract specific crystallographic information.* ***g,*** *inverse FFT of the spatial frequency mask, illustrating the atomic arrangement of Sn.* ***h,*** *atomic visualisation model of the [010] tetragonal β-phase of Sn, showing alignment with the experimentally observed crystal structure. Scale bar:* ***a****, 5 nm,* ***b*** *&* ***c****, 3 nm,* ***d*** *&* ***g****, 0.5 Å, and* ***e*** *&* ***f****, 3.5 nm^{-1}.*

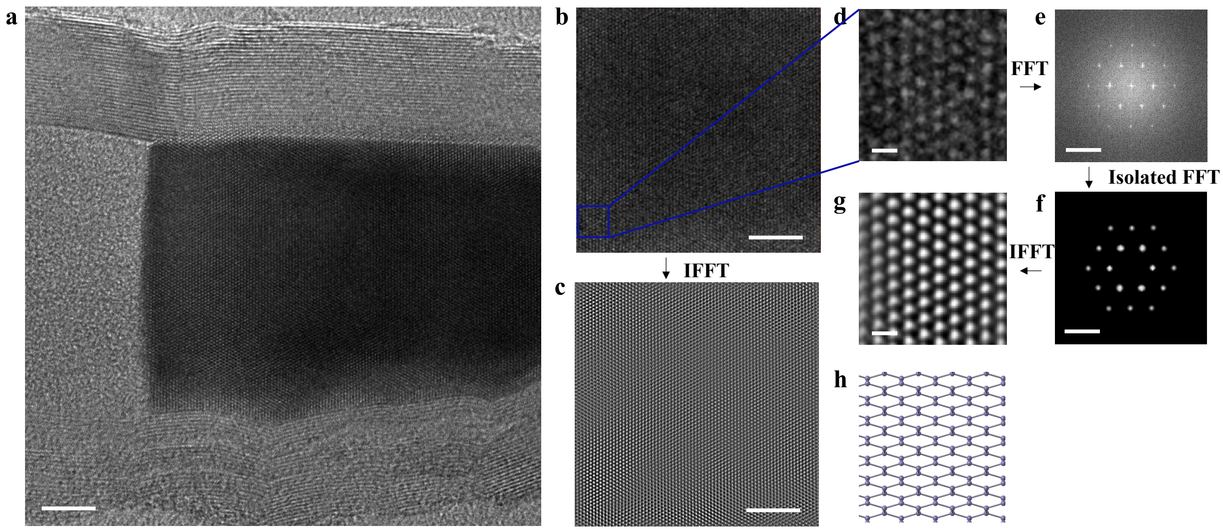

Suppl. Fig. 2: Tetragonal β-phase of a Sn nanowire encapsulated within a MWCNT imaged along the [001] zone axis. a, *bright-field atomic-resolution TEM (BF-ARTEM) micrograph showing the single-crystal nature of the encapsulated Sn nanowire.* ***b,*** *cropped region of the ARTEM micrograph (blue box inset), selected for detailed analysis.* ***c,*** *inverse fast Fourier transform (iFFT) of the cropped ARTEM micrograph, highlighting the positions of Sn atoms across the nanowire.* ***d,*** *magnified ARTEM micrograph displaying the tetragonal crystal face of β-Sn.* ***e,*** *Fast Fourier transform (FFT) of the magnified ARTEM micrograph.* ***f,*** *isolated mask of the FFT spatial frequencies, used to extract specific crystallographic information.* ***g,*** *inverse FFT of the spatial frequency mask, illustrating the atomic arrangement of Sn.* ***h,*** *atomic visualisation model of the [010] tetragonal β-phase of Sn, showing alignment with the experimentally observed crystal structure. Scale bar:* ***a-c****, 5 nm,* ***d*** *&* ***g****, 5 Å, and* ***e*** *&* ***f****, 7 nm*$^{-1}$.

The interplanar *d*-spacings of encapsulated Sn nanowires were determined from Fast Fourier Transform (FFT) patterns obtained from ARTEM micrographs. These FFTs capture the spatial frequency of the periodic atomic lattice and correspond to the reciprocal lattice of the imaged zone axis. The spatial resolution was estimated from spot broadening of the FFT spots, yielding uncertainties of ± 0.35 nm^{-1} for the tetragonal reflections and ± 0.378 nm^{-1} and ± 0.326 nm^{-1} for cubic reflections. Comparison between the measured *d*-spacings and bulk β-Sn is presented in Supplementary Table S1 and Table S2 below for both the cubic and tetragonal crystal face, respectively. The reference reflection list was generated by modelling the β-Sn structure (space group $I4_1/amd$; a = 5.83 Å, c = 3.18 Å) in CrystalMaker and simulating the powder X-ray Diffraction (XRD) pattern to extract expected reflection positions and intensities. Minor deviations between measured and reference *d*-spacings may arise from local strain in the encapsulated Sn, projection effects, or imaging aberrations.

Suppl. Table 1: Measured and reference interplanar *d*-spacings for encapsulated β-Sn nanowire viewed down the cubic [001] zone axis.

assigned plane	FFT freq. (nm^{-1})	FFT ± Δfreq. (nm^{-1})	measured d (Å)	± Δd (Å)	literature d (Å)	Δd (Å) = meas – lit	deviation (%)
**(4 0 0)**	6.819	0.326	1.466	±0.070	1.458	0.008	0.57
**(4 2 0)**	7.317	0.378	1.366	±0.071	1.304	0.062	4.76

Suppl. Table 2: Measured and reference interplanar *d*-spacings for encapsulated β-Sn nanowire viewed down the tetragonal [010] zone axis.

assigned plane	FFT freq. (nm^{-1})	FFT ± Δfreq. (nm^{-1})	measured d (Å)	± Δd (Å)	literature d (Å)	Δd (Å) = meas – lit	deviation (%)
**(3 0 1)**	3.595	0.35	2.782	±0.27	2.794	−0.012	-0.43
**(2 0 0)**	3.468	0.35	2.883	±0.29	2.945	−0.062	-2.11

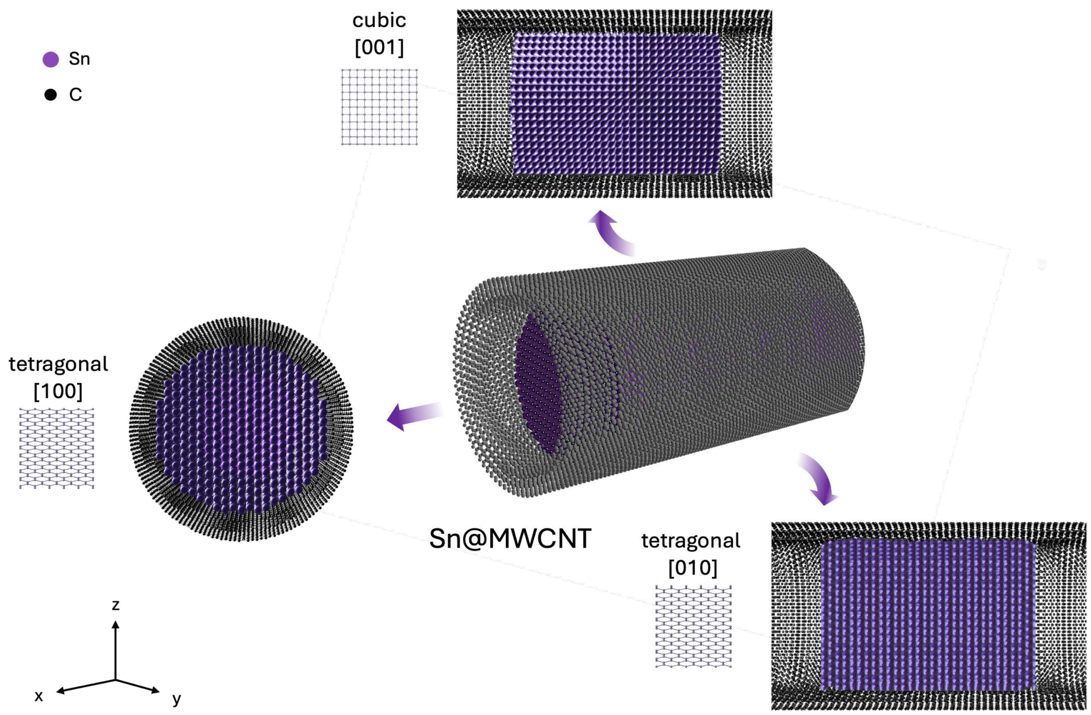

Suppl. Fig. 3: Orientation analysis of two encapsulated Sn nanowires. *Schematic illustration showing the crystallographic orientation and spatial configuration of two single-crystal β-Sn nanowires confined within a MWCNT. The cubic [001] and tetragonal [010] faces are observed in contact with the inner walls of the nanotube, while the tetragonal [100] face lies along the nanotube axis. This configuration highlights the orthogonal relationship between crystal facets and the cylindrical confinement. Face assignment was based on the observed faceting at the interface between the nanowire and the CNT wall, indicating that both nanowires are oriented with the [001] and [010] faces facing the wall, and the [100] face directed along the axial channel of the nanotube. Purple atoms denote Sn, while black atoms represent carbon atoms forming the MWCNT wall. The inner diameter of the MWCNT is 3 nm.*

2. Disproportionation behaviour of SnO

The amorphous character of the encapsulated material inside the nanotube arises from the complex thermochemistry of SnO, which undergoes multi-step disproportionation involving intermediate oxidation states. This transformation can be broadly represented as:

$$2\mathrm{SnO}_{(s)} \rightarrow \text{intermediate } \mathrm{Sn_xO}_{(g)} \rightarrow \mathrm{Sn}_{(s)} + \mathrm{SnO}_{2\,(s)}$$

where the intermediate vapour-phase species (Sn_xO) may include mixed-valence compounds, such as Sn_2O_3 and Sn_3O_4 – both of which were observed as transient crystalline domains *in situ*, see Supplementary Section 5. The formation and decomposition of these intermediates are governed by the local oxygen chemical potential and vapour saturation within the confined nanotube environment. This dynamic evolution of phases disrupts long-range crystallinity, producing an amorphous matrix interspersed with Sn-rich and Sn-poor regions. The resulting heterogeneity reflects the reactive nature of SnO disproportionation and its role in driving the encapsulation process under confinement.

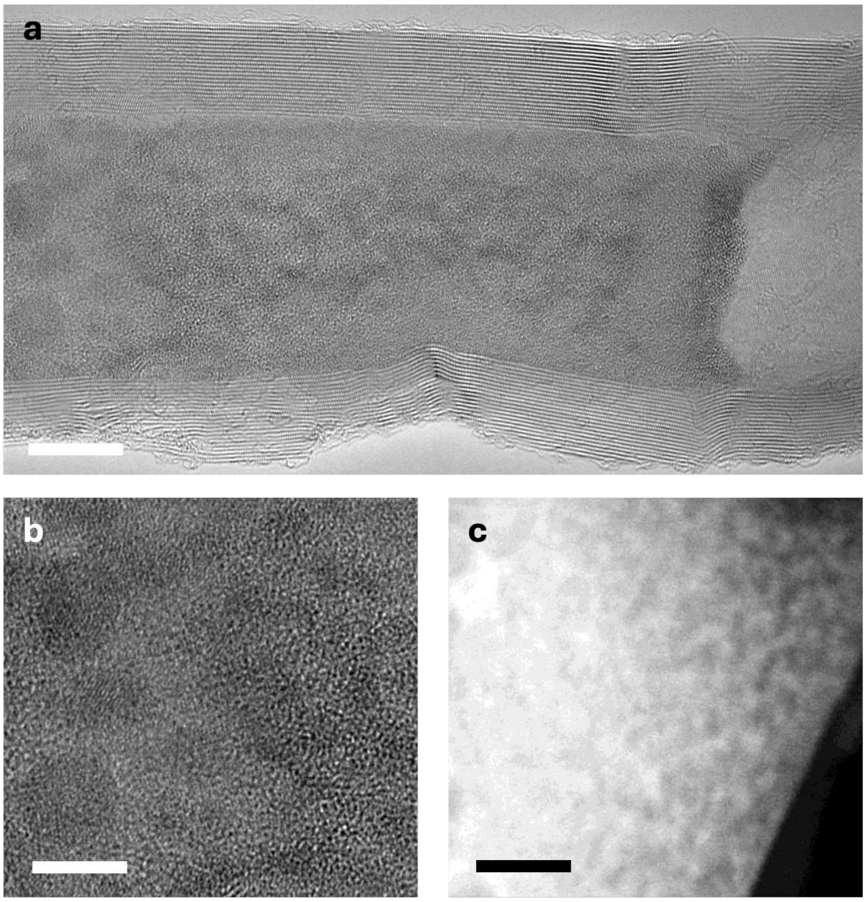

***Suppl. Fig. 4: ARTEM image of disproportionation of Sn_xO within the nanotube core. a**, Bright-field ARTEM micrograph showing a Sn_xO-filled MWCNT. **b**, High-magnification ARTEM micrograph highlighting the amorphous morphology of the encapsulated Sn_xO nanowire, showing the local disproportionation, with dark contrasting regions corresponding to metallic Sn embedded*

within an amorphous oxide matrix. ***c****, Angular dark field-scanning transmission electron microscopy micrograph of a similar region, complementing the bright-field ARTEM micrograph, showing chemical contrast with the bright metallic Sn domains embedded within the oxide matrix, providing insight into local disproportionation upon cooling. Scale bars:* ***a****, 10nm,* ***b****, 4 nm, and* ***c****, 5 nm.*

A representative bright-field ARTEM micrograph and annular dark-field - scanning transmission electron microscopy (ADF-STEM) image (Suppl. Fig. 4) reveal an amorphous, oxygen-rich Sn_xO domain encapsulated within the central cavity of a MWCNT. Embedded within this matrix are nanocrystalline domains ranging from 1–4 nm, displaying heterogeneous contrast in both imaging modes. This variation likely reflects local Sn enrichment, as regions with higher atomic number (Z) scatter electrons more strongly, producing brighter or darker features depending on the imaging conditions.

SnₓO@MWCNT

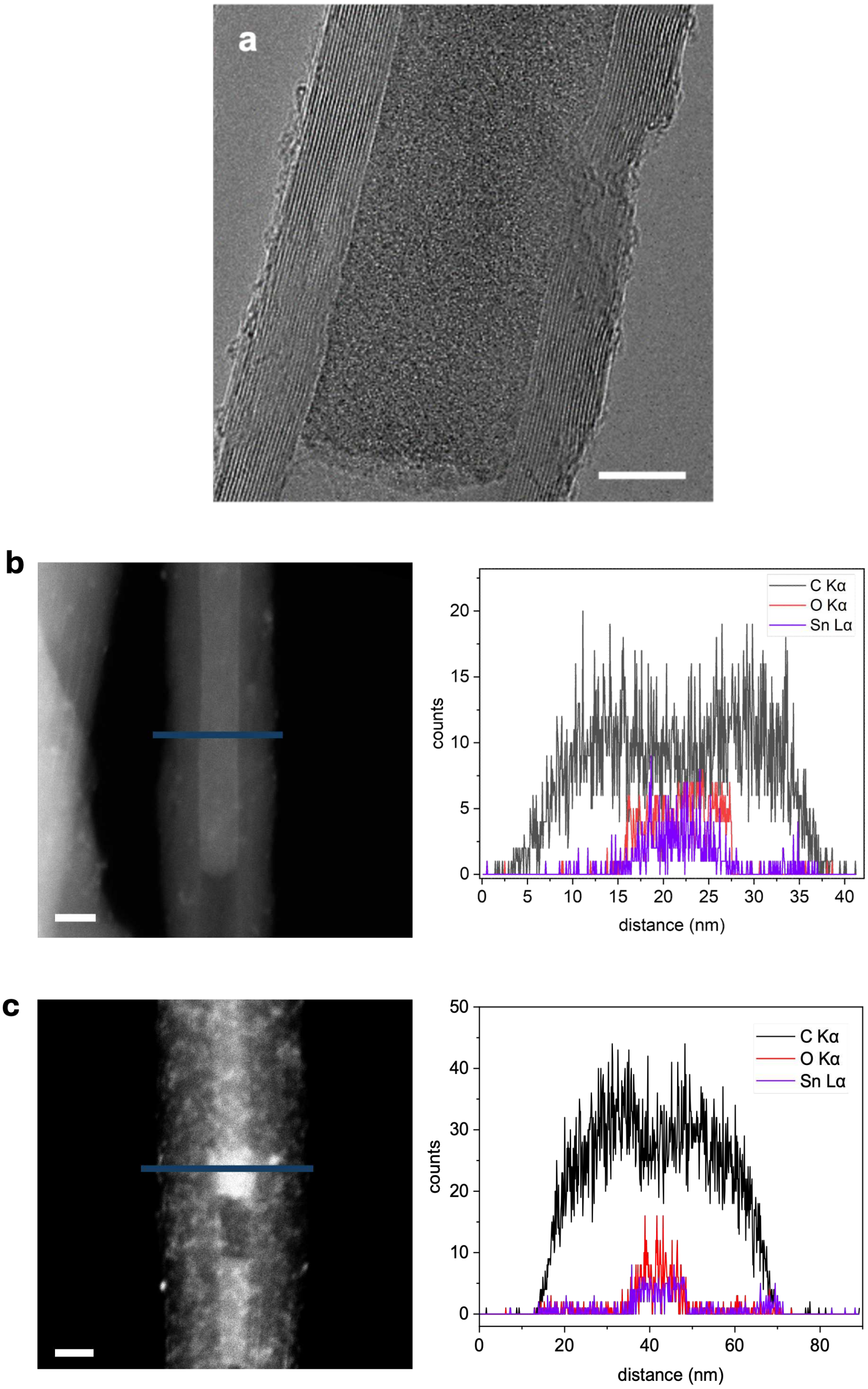

Suppl. Fig. 5: Energy dispersive X-ray spectroscopy (EDS) confirming the encapsulation of Sn_xO within MWCNTs. ***a****, Bright-field-ARTEM micrograph of Sn_xO-filled MWCNT (Sn_xO@MWCNT).* ***b & c****, ADF-STEM micrographs of Sn_xO-filled WMCNTs with the associated EDS linescan profiles acquired across the Sn_xO-filled nanotube diameter, showing spatially resolved Sn and O signals confined to the inner core, confirming the axial encapsulation of Sn_xO.*

EDS measurements were performed at 80 kV under beam-sensitive conditions. Scale bar: ***a****, 10 nm,* ***b****, 10 nm,* ***c****, 10 nm.*

To confirm the composition of the encapsulated phase, elemental analysis was conducted using Energy-Dispersive X-ray Spectroscopy (EDS) line scans across the MWCNT. The simultaneous presence of Sn and O peaks, spatially centred within the carbon shell, confirms the axial confinement of Sn_xO within the nanotube.

3. Convolution Neural Network (CNN) micrograph processing:

To automate the pixel-wise classification of high-resolution ARTEM micrographs, we developed a CNN-based micrograph processing pipeline that preserves atomic-scale details. Our workflow encompasses micrograph acquisition, manual labelling for CNN training, and pixel-level classification, ensuring that the original resolution and precise atom placements are maintained throughout processing. The following sections describe the complete workflow in detail.

Micrograph training

Depending on the experiment, between 19 and 22 training micrographs were collected, with a micrograph size ranging from approximately 1500×2500 to 2500×5000 pixels. The micrographs were stored as 8-bit grayscale TIFF files to avoid compression artefacts. Each training micrograph was manually annotated at the pixel level to label seven distinct atomic features: MWCNT wall, empty tube, amorphous Sn_xO, liquid Sn_xO, Sn_2O_3 (metallic oxide), Sn_3O_4, and background. The micrographs were cropped to predominantly include the MWCNT region and rotated so that the nanotubes were vertically aligned. Suppl. Fig. 6 shows a sample nanotube micrograph along with its corresponding expert annotations, with different colours representing the various regions. During acquisition, care was taken to ensure that each pixel area corresponded to the same physical area across all micrograph samples.

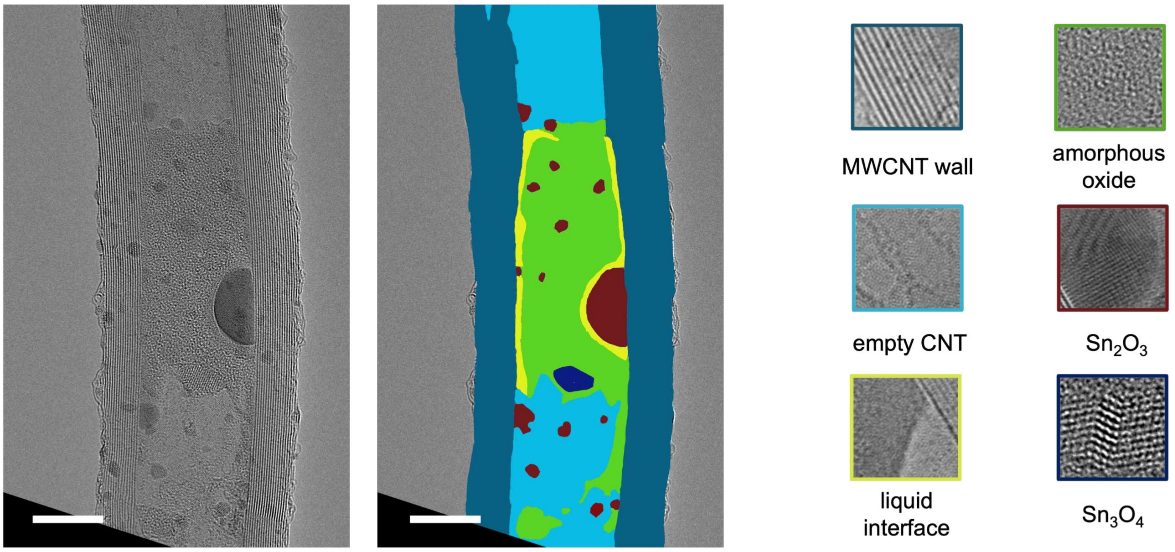

Suppl. Fig. 6: ARTEM micrograph labelling. *Example of ARTEM micrograph labelling for CNN training. The left panel shows an original ARTEM micrograph alongside a corresponding manually labelled micrograph. The right panel displays the corresponding regions according to each pixel class. Scale bar:* ***a****, 10 nm,* ***b****, 10 nm.*

Model

Due to the limited number of micrographs and their extreme spatial resolution, which pose computational challenges for pixel-wise region classification, a CNN patch filter was implemented. Specifically, the CNN was trained to classify each pixel based on the surrounding micrograph patch of size 64×64 pixels. In TEM micrographs, where key features such as atomic columns, lattice fringes, and phase boundaries often span only a few pixels, this loss proved detrimental. In contrast, our patch-based CNN performs pixel-wise classification directly at the full resolution without interpolation, ensuring that fine details essential for accurate morphological and defect analysis are

retained. This approach is particularly beneficial for dynamic *in situ* studies where transient and localised phenomena, such as single atom movement, may only be evident when observed at the native resolution.

The neural network employed in this study is a convolutional encoder with skip connections to classify pixels to region types based on input patches. The network architecture (Suppl. Fig. 7) comprised four residual blocks, each followed by a max-pooling layer for downsampling. Each residual block contained two convolutional layers and a skip connection. A single convolutional layer preceded the first residual block, while two dense layers followed the last, with sizes of 256 and N (where N represents the number of considered nanotube region types). All convolutions used 64 filters of size 3 x 3 pixels, and Leaky ReLU activation was applied after each convolutional layer. Finally, a softmax activation function was used to generate class probabilities.

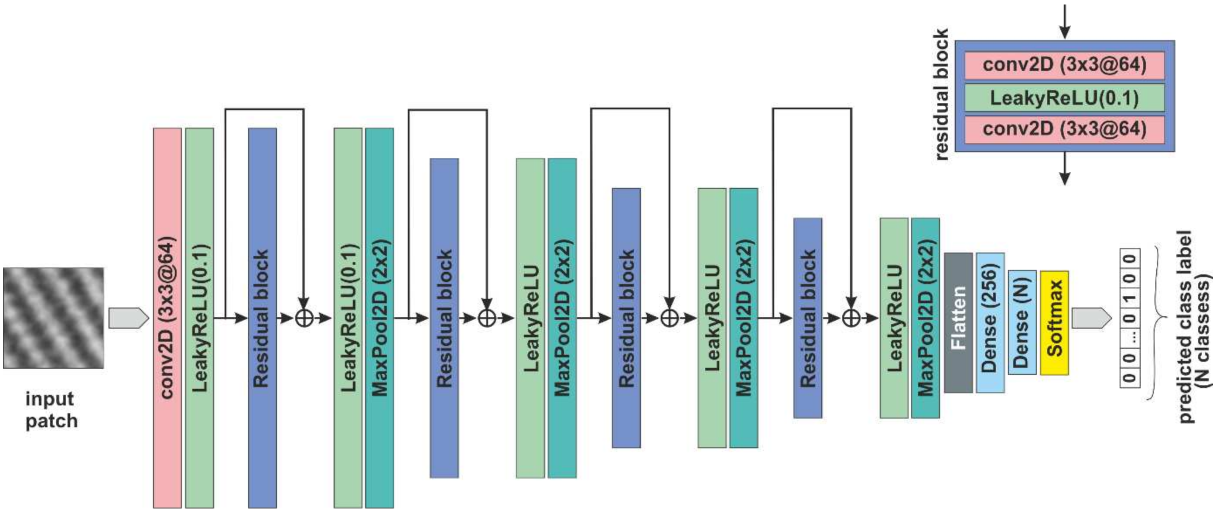

Suppl. Fig. 7: Schematic of the CNN architecture employed. *The network accepts a 64×64 input patch, passes it through an initial 3×3 convolutional layer (64 filters), and then through multiple residual blocks with LeakyReLU activations. The final layer is a fully connected layer for classification.*

Patches extraction for training

The CNN model was trained on 64 × 64 pixel patches extracted from ARTEM micrographs. Patches were systematically sampled row-by-row and column-by-column with a 10-pixel stride, ensuring that each patch contained at least one labelled pixel to reduce the prevalence of background regions. This approach generated approximately 35,000 to 120,000 training patches per micrograph, depending on the specific experiment and micrograph resolution. Each patch was assigned a region label based on the central pixel, capturing local structural features for robust feature learning. Prior to training, micrograph intensities were normalised to the range [0, 1] by dividing pixel values by 255 to standardise input contrast. The dataset was split into training (80%) and validation (20%) subsets to assess model performance. Representative examples of the extracted patches, covering the range of considered region types, are shown in Suppl. Fig. 8.

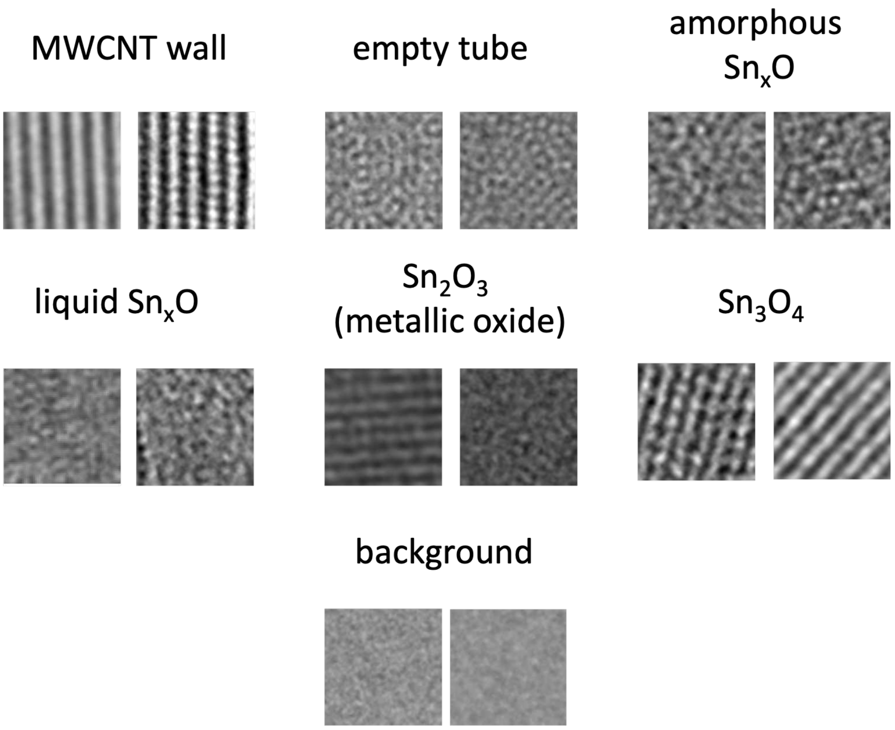

Suppl. Fig. 8: Representative Training Patches for Pixel Classification. *Examples of sample training patches (64 x 64 pixels; 2 nm x 2 nm) representing considered regions across the seven class types.*

Model training

The convolutional neural network was trained for 200 epochs using a mini-batch size of 4096. The Adam optimiser, configured with an initial learning rate of 0.001 and a decay rate of 0.001, was employed to minimise the cross-entropy loss. Early stopping was applied with a patience of 20 epochs, halting training after 20 consecutive epochs without improvement in the validation loss. The model weights corresponding to the lowest validation loss were then selected for inference. All training was performed on an NVIDIA Quadro RTX 6000 GPU.

Handling micrograph variance in ARTEM training

ARTEM micrographs exhibit significant contrast variance, as the micrograph intensity depends on the interference of electron waves that pass through and exit the sample. This phase contrast is highly sensitive to experimental parameters including defocus, sample thickness, and beam brightness, which can alter the appearance of atomic scale features, making them appear either brighter or darker depending on imaging conditions. These factors introduce artefacts that introduce variance in feature extraction and complicate classification in CNN recognition.

To address this, our CNN model was trained on a diverse set of 64 × 64 pixel patches extracted from micrographs collected under varying experimental conditions. This patch-based approach not only captures localised structural information but also reduces the influence of global contrast shifts, beam intensity fluctuations, and thickness variations. By exposing the model to a wide range of imaging conditions, we ensure that it learns to identify physically meaningful structures, improving classification accuracy and robustness against noise and artefacts. This strategy broadens the generalisation of the model to new, unseen data, to capture the underlying atomic features of the patches.

Pixel classification

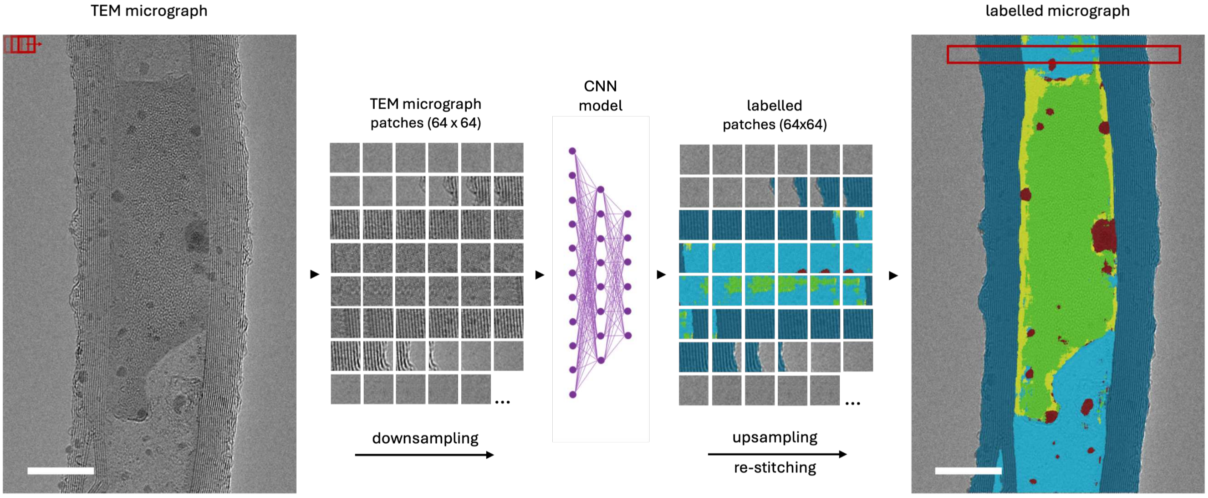

Suppl. Fig. 9: Patch-based segmentation strategy used to annotate the ARTEM micrographs whilst reattaining the original resolution of the micrograph. *The framework is based on the segmentation of the micrograph into numerous 64 x 64 pixel patches, where the central pixel is assigned by processing the patch through the pre-trained CNN model, and re-stitched through upsampling and overlaying of the patches to construct the fully annotated TEM micrograph. Scale bar: 10 nm.*

The trained CNN was used to classify consecutive pixels in unseen TEM micrograph. A sliding window approach was employed, where a patch-sized window was moved across the micrograph row-by-row and column-by-column. The framework of the patch-based strategy is shown in Suppl. Fig. 9 by the rastering of a 64 x 64 pixel patch across the micrograph that are classified through the model and reconstructed into a complete micrograph whilst retaining the spatial classification. Patches were sampled with a stride of 1, and the predicted label for each patch was assigned to its centre pixel. This process produced a continuous region-label map, where labels were assigned to every pixel in the micrograph.

4. *In situ* ARTEM and CNN processed micrographs:

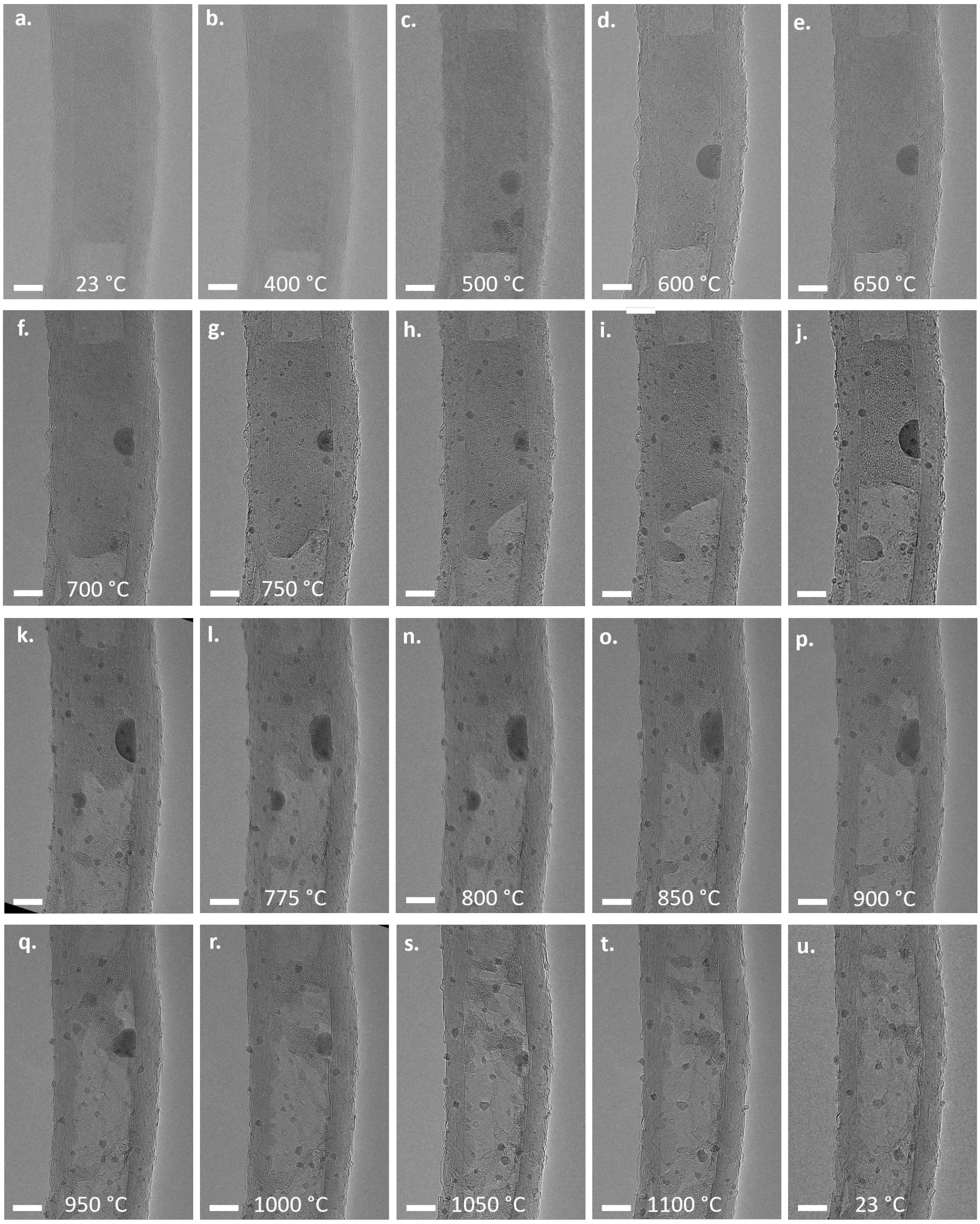

Suppl. Fig. 10: Corresponding in situ time series of unlabeled ARTEM micrographs as shown in figure 2 b–u. *These untrained micrographs are processed by the CNN model. All micrographs were captured at 80 kV. Morphological evolution of Sn_3O_4 at various temperatures:* ***a****, static conditions 23 °C,* ***b,*** *400 °C* ***c****, 500 °C,* ***d****, 600 °C,* ***e****, 650 °C,* ***f****, 700 °C,* ***g – k****, 750 °C,* ***i,*** *775 °C* ***n****, 800 °C,* ***n****, 900 °C,* ***o****, 1000 °C,* ***p****, 1050 °C,* ***q****, a static micrograph upon cooling at 23 °C. Scale bar:* ***a-q****, 10 nm.*

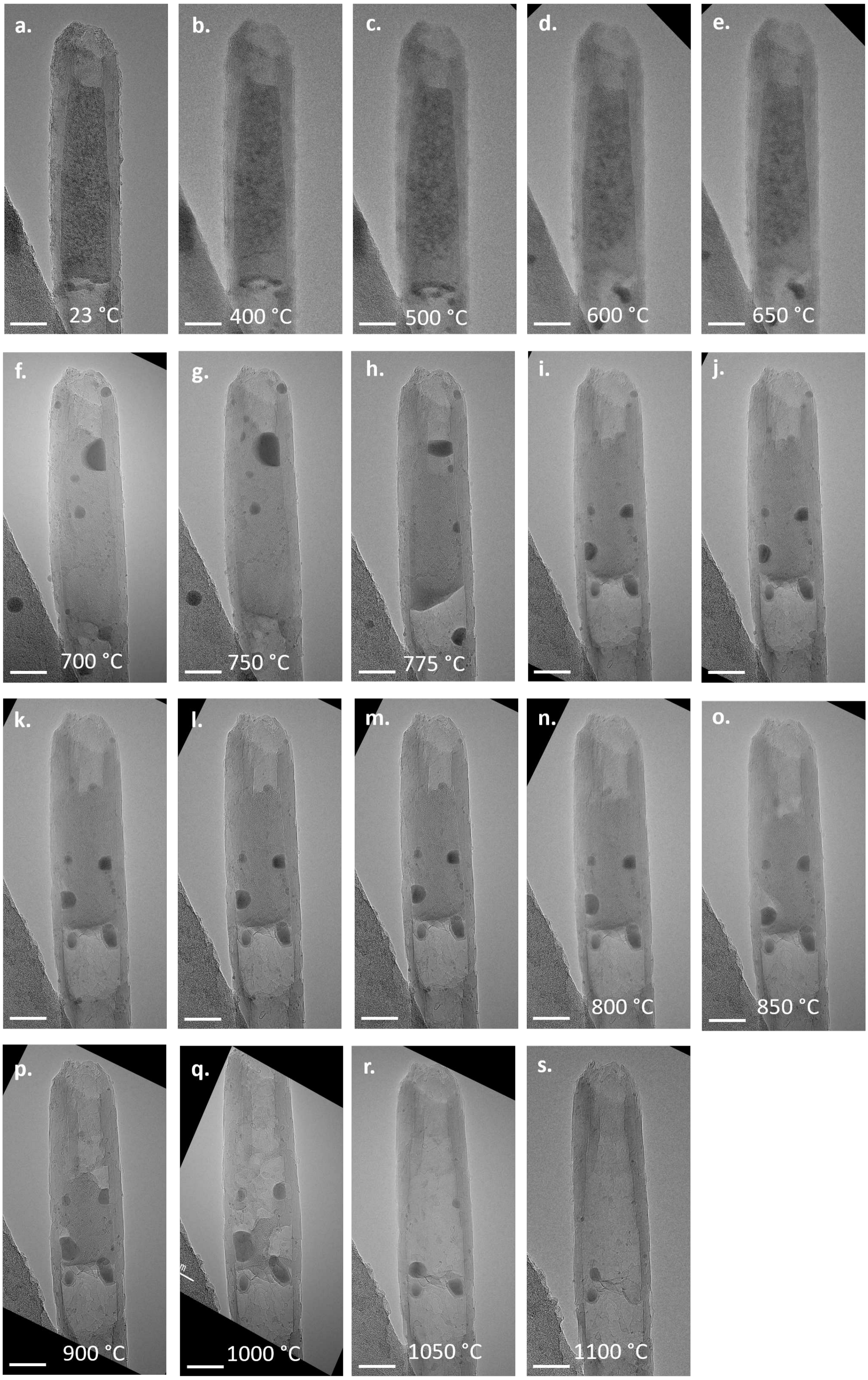

Suppl. Fig. 11: Unprocessed in situ BF-ARTEM micrographs of a second encapsulated Sn_xO nanowire encapsulated within a MWCNT. *All micrographs were captured at 80 kV.* ***a,*** *under static conditions at 23 ℃,* ***b,*** *400 ℃,* ***c,*** *500 ℃,* ***d,*** *600 ℃,* ***e,*** *650 ℃,* ***f,*** *700 ℃,* ***g,*** *750 ℃,* ***h – m,*** *775 ℃,* ***n,*** *800 ℃,* ***o,*** *850 ℃,* ***p,*** *900 ℃,* ***q,*** *1000 ℃,* ***r,*** *1050 ℃,* ***s,*** *1100 ℃. Scale bar:* ***a-s****, 20 nm.*

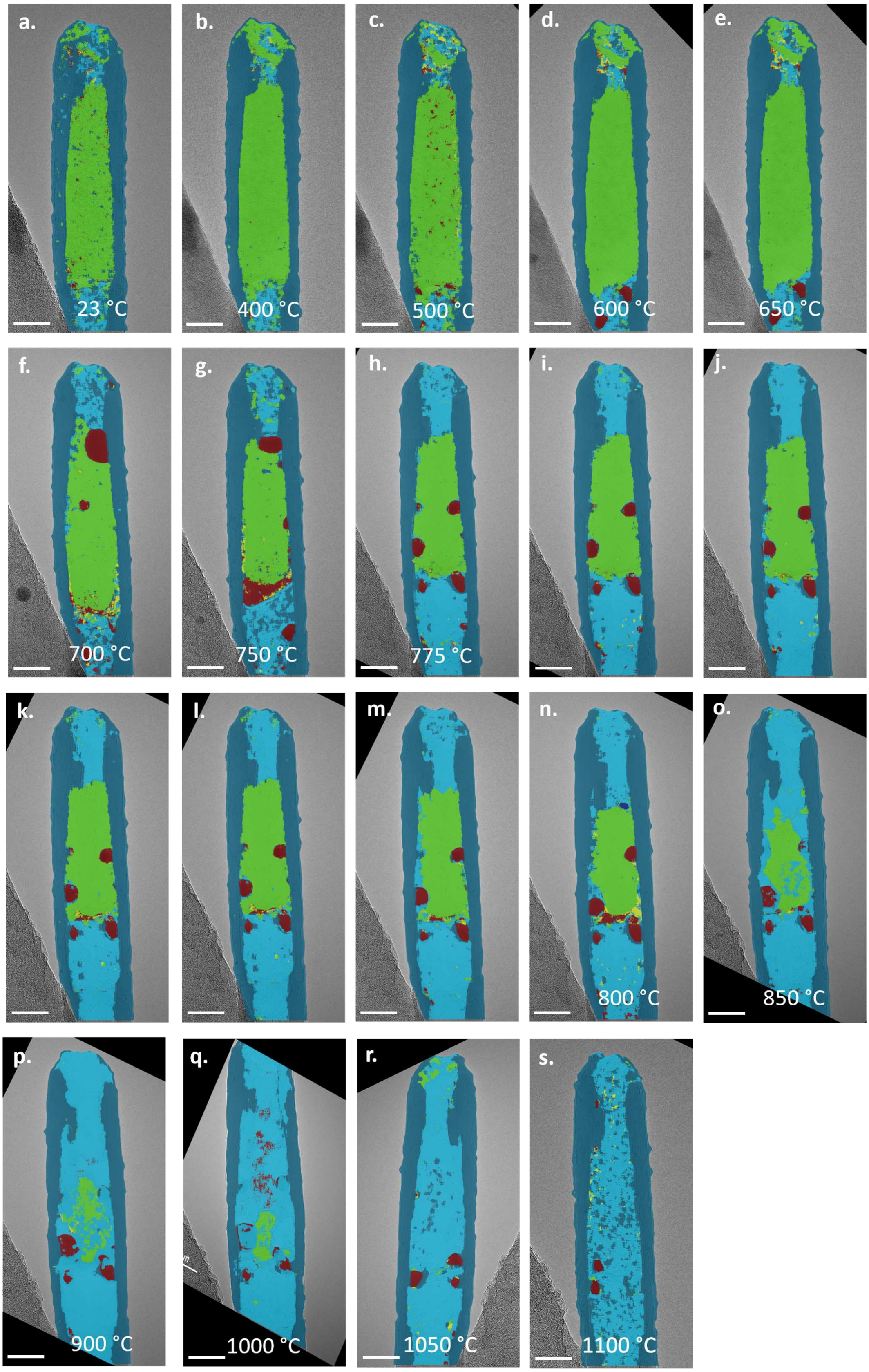

Suppl. Fig. 12: Corresponding CNN-labelled micrographs of the second encapsulated Sn_xO nanowire encapsulated within a MWCNT. a, *under static conditions at 23 ℃,* ***b,*** *400 ℃,* ***c,*** *500 ℃,* ***d,*** *600 ℃,* ***e,*** *650 ℃,* ***f,*** *700 ℃,* ***g,*** *750 ℃,* ***h–m,*** *775 ℃,* ***n,*** *800 ℃,* ***o,*** *850 ℃,* ***p,*** *900 ℃,*

q, *1000 ℃,* ***r,*** *1050 ℃,* ***s,*** *1100 ℃. Colour-coded labels indicate textural classes identified by the CNN: blue (MWCNT wall), light blue (empty MWCNT core), green (amorphous Sn_xO), dark red (metallic Sn_2O_3), and yellow (liquid-phase Sn_xO). Scale bar:* ***a***-***s****, 20 nm.*

5. *In situ* formation of Sn_3O_4 and Sn_2O_3:

In situ observations reveal that intermediate Sn_xO nanowires form during the early stages of vaporisation, subsequently undergoing disproportionation into transient mixed-valence phases such as Sn_2O_3 and Sn_3O_4 phases within the confined nanotube core, at various temperatures. These intermediates emerge at elevated temperatures and are likely derived from vapour-phase species, reflecting the complex phase evolution under confinement.

Triclinic Sn_2O_3 at 600 °C

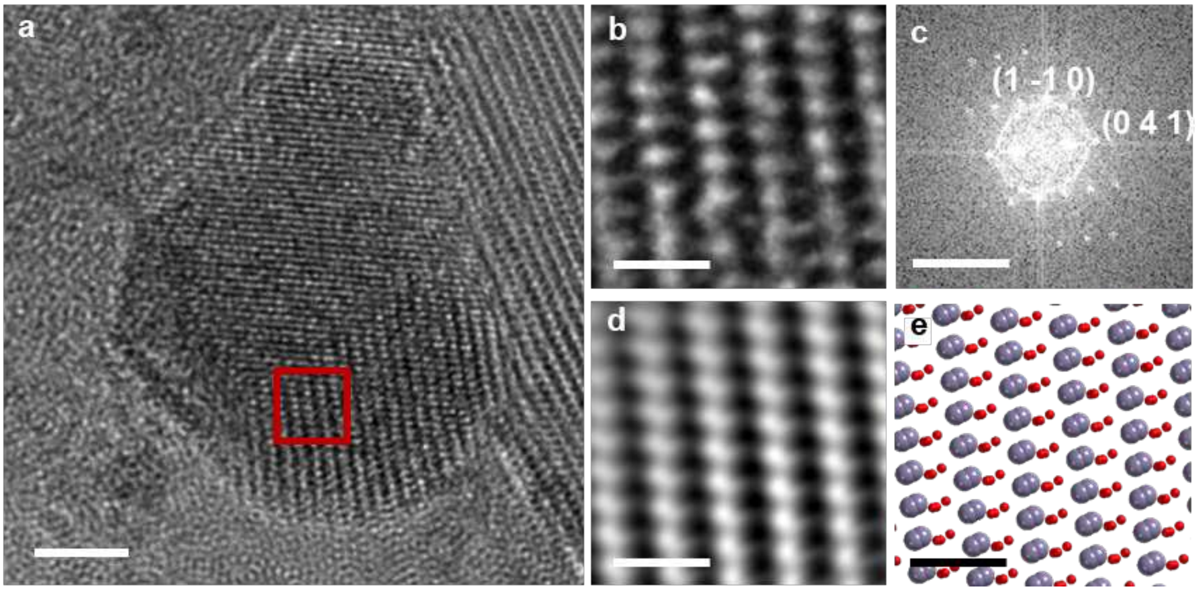

***Suppl. Fig. 13: in situ formation of intermediate Sn_2O_3 oxide imaged at 600 °C. a**, Bright-field-ARTEM micrograph providing an overview of the Sn_2O_3 crystal that formed at the carbon surface. **b**, A high-resolution image captures the atomic structure along the [-1 1 5] zone axis, where linear columns of bright contrast corresponding to Sn atoms are visible. **c**, The FFT reveals distinct spatial frequencies corresponding to the (1 -4 1) and (1 1 0) planes of Sn_2O_3. **d**, An inverse-FFT reconstructs the real-space atomic distribution, further confirming lattice periodicity. **e**, simulated [-1 1 5] face viewed under ARTEM. Purple atoms represent Sn atoms, and red represent oxygen atoms. Scale bar: **a**, 2 nm, **b**, 0.7 nm, **c**, 9 nm^{-1} **d**, 0.7 nm.*

The crystal structure of Sn_2O_3 was identified by analysing the orientation and atomic arrangement in the ARTEM images. Using experimental X-ray diffraction data from literature[1], the unit cell was modelled with parameters: a = 3.710 Å, b = 8.180 Å, c = 5.460 Å; $\alpha = 90.000°$, $\beta = 92.300°$, and $\gamma = 93.800°$. Simulations were conducted using CrystalMaker to calculate *d*-spacings and generate atomic projections along the [1 1 −4] zone axis. A comparison between the experimentally measured and simulated *d*-spacings (Suppl. Table 3) confirms the identification of the triclinic Sn_2O_3 phase. The slight deviation from ideal lattice vectors suggests a distorted superlattice structure, consistent with the observed metallic contrast and crystallographic distortions.

Suppl. Table 3: Assigned planes and measured *d*-spacings of the Sn_2O_3 intermediate oxide observed in Suppl. Fig. 13 imaged at 600 °C.

assigned plane	FFT freq. (nm^{-1})	FFT ± Δfreq. (nm^{-1})	measured d (Å)	± Δd (Å)	literature d (Å)	Δd (Å) = meas – lit	deviation (%)
**(1 -4 1)**	5.79	0.44	1.73	0.013	1.733	-0.003	-0.17
**(1 1 0)**	3.03	0.46	3.30	0.050	3.288	0.012	0.36

Throughout the *in situ* heating series, triclinic Sn_2O_3 phases were consistently with clearly resolved lattice fringes from 600-900°C. Similarly, Suppl. Fig. 14 presents a structural analysis of a triclinic Sn_2O_3 phase observed at 900 °C, further supporting the presence of this phase at elevated temperatures. A corresponding comparison of measured and simulated lattice spacings (Suppl. Table 4).

Triclinic Sn_2O_3 at 900 °C

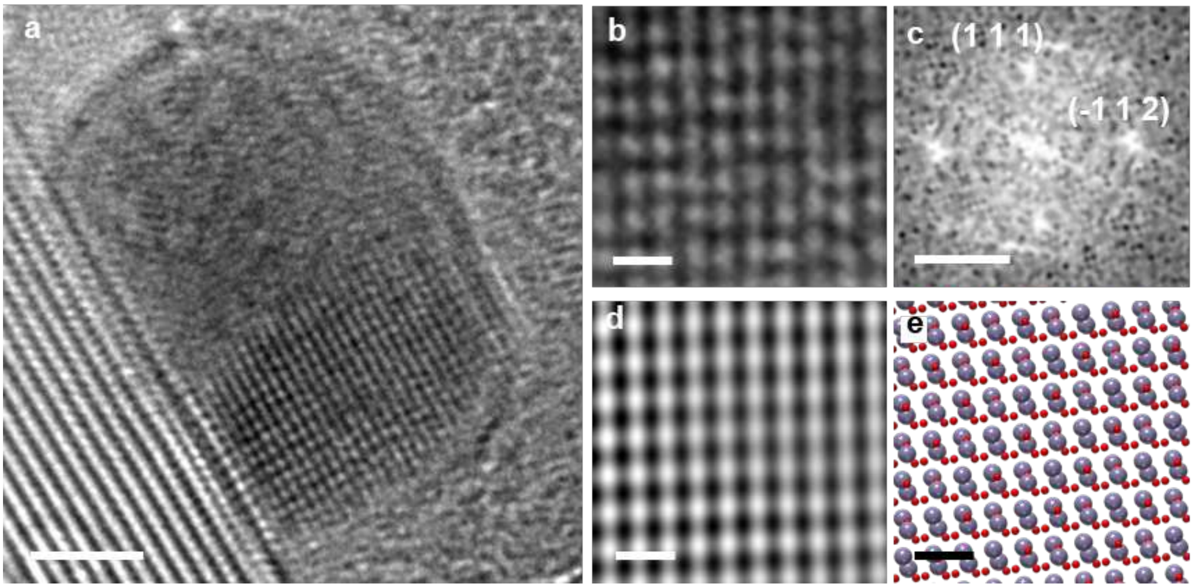

***Suppl. Fig. 14: Structure of the triclinic Sn_2O_3 crystal imaged at 900 °C. a**, Low-magnification ARTEM micrograph of Sn_2O_3 crystal structure. **b**, High-magnification ARTEM micrograph imaged down the atomic columns of Sn_2O_3, **c**, Fast Fourier Transform (FFT) indicating the spatial distribution of periodic array, **d**, inverse-FFT (iFFT) of the isolate spatial frequencies that reveal the atomic array of the [1 -3 2] face. e, simulated projection of the [1 -3 2] face for comparison. Purple atoms represent the Sn atoms, and oxygen atom represented in red. Scale bar: **a**, 2 nm, **b**, 0.7 nm, **c**, 9 nm^{-1}, **d**, 0.7 nm.*

Suppl. Table 4: Measured and calculated *d*-spacings used for plane assignments for triclinic Sn_2O_3 at 900°C as observed in Suppl. Fig. 14.

assigned plane	FFT freq. (nm^{-1})	FFT ± Δfreq. (nm^{-1})	measured d (Å)	± Δd (Å)	literature d (Å)	Δd (Å) = meas – lit	deviation (%)
**(1 1 1)**	7.28	0.46	2.79	0.087	2.77	-0.03	-1.08
**(-1 1 2)**	9.17	0.46	2.18	0.058	2.18	0.00	0.00

Sn_3O_4 at 750 °C

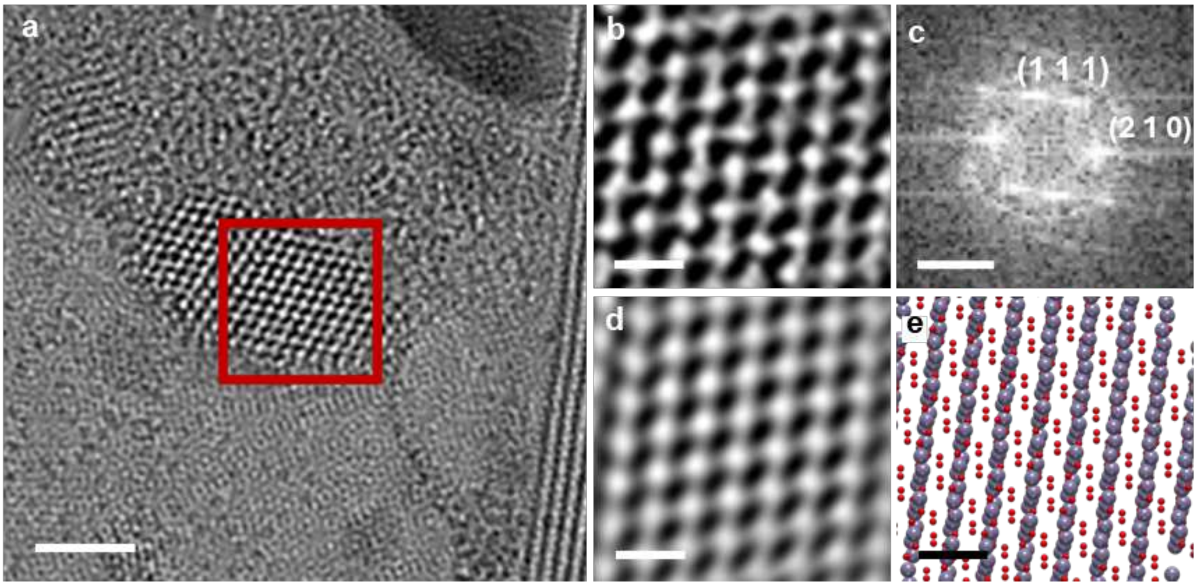

Suppl. Fig. 15: Direct crystallographic evidence of Sn_3O_4 speciation. ***a***, *Overview ARTEM micrograph showing the Sn_3O_4 crystal structure at the liquid interface, recorded at 750°C.* ***b***, *ARTEM micrograph of the Sn_3O_4 [-1 2 -1] zone axis,* ***c***, *Fast Fourier Transform (FFT) displaying the spatial distribution corresponding to the (111) and (210) planes.* ***d***, *inverse-FFT (iFFT) reconstructed using the (111) and (210) spatial frequencies, used to map the atomic positions.* ***e***, *Simulated Sn_3O_4 structure oriented to the [-1 2 -1] face, illustrating the alignment of Sn atomic columns and interstitial positioning of oxygen atoms. Purple atoms represent Sn, and red represents the oxygen atoms. Scale bar:* ***a***, *3 nm,* ***b***, *0.5 nm,* ***c***, *5 nm^{-1},* ***d***, *0.5 nm,* ***e***, *0.5 nm.*

During the *in situ* vaporisation of the Sn_xO nanowire, Figure S11 shows the formation of an Sn_3O_4 crystal at 750 °C at the liquid-vapour interface. This short-lived intermediate, which vaporises within a few frames, is consistent with the monoclinic $P2_1/c$ structure, representing a distorted superlattice variant of cassiterite SnO_2 as reported[2]. The measured *d*-spacings and corresponding crystal planes for this Sn_3O_4 intermediate, extracted from FFT analysis, are presented in Suppl. Table 5.

Suppl. Table 5: Assigned planes and measured *d*-spacings of the Sn_3O_4 intermediate oxide observed in Suppl. Fig. 15 imaged at 750 °C.

assigned plane	FFT freq. (nm^{-1})	FFT ± Δfreq. (nm^{-1})	measured d (Å)	± Δd (Å)	literature d (Å)	Δd (Å) = meas – lit	deviation (%)
(1 -4 1)	5.79	0.44	1.73	0.013	1.733	-0.003	-0.17
(1 1 0)	3.03	0.46	3.30	0.050	3.288	0.012	0.36

6. Angular dark-field - scanning transmission electron microscopy (ADF-STEM)

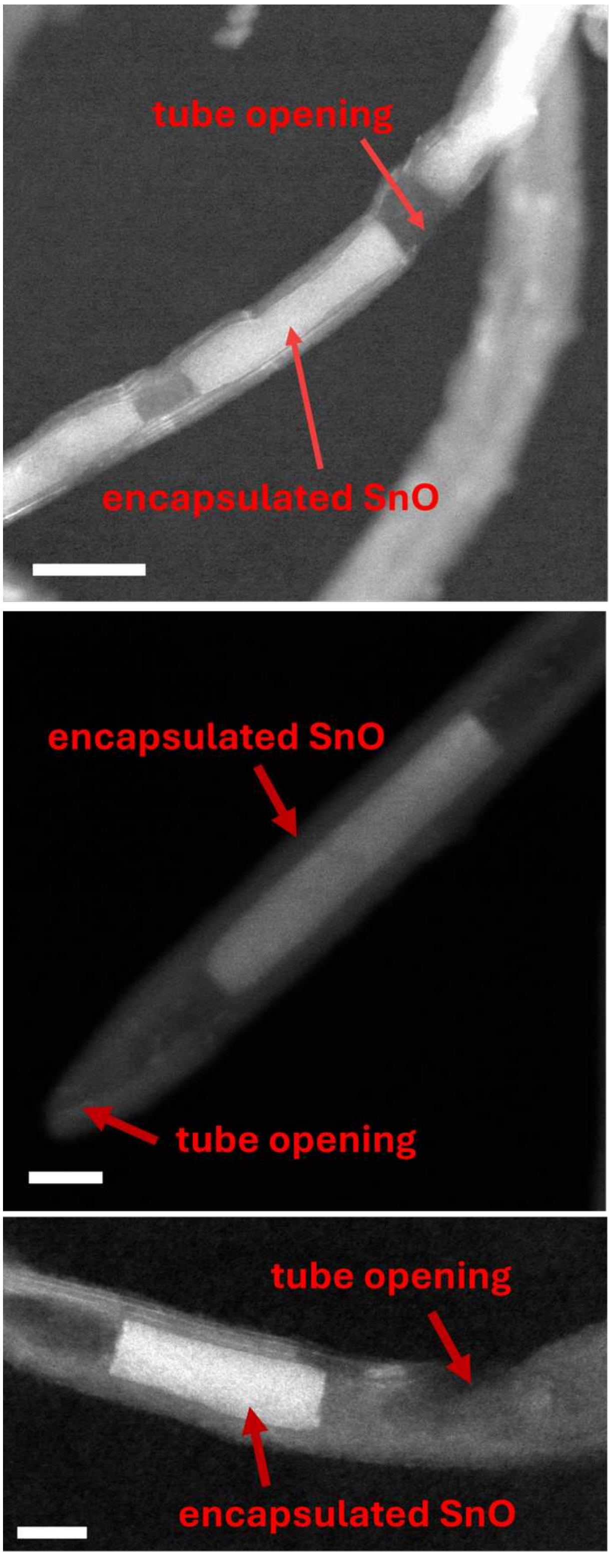

Suppl. Fig. 16: Location analysis of Sn_xO nanowires to the tube opening. *Angular dark-field-scanning transmission electron microscopy (ADF-STEM) micrographs depicting Sn_xO nanowires (bright contrast) encapsulated within an MWCNT, illustrating droplet condensation near the tube entrance.*

7. Electron Energy Loss (EELS) Analysis

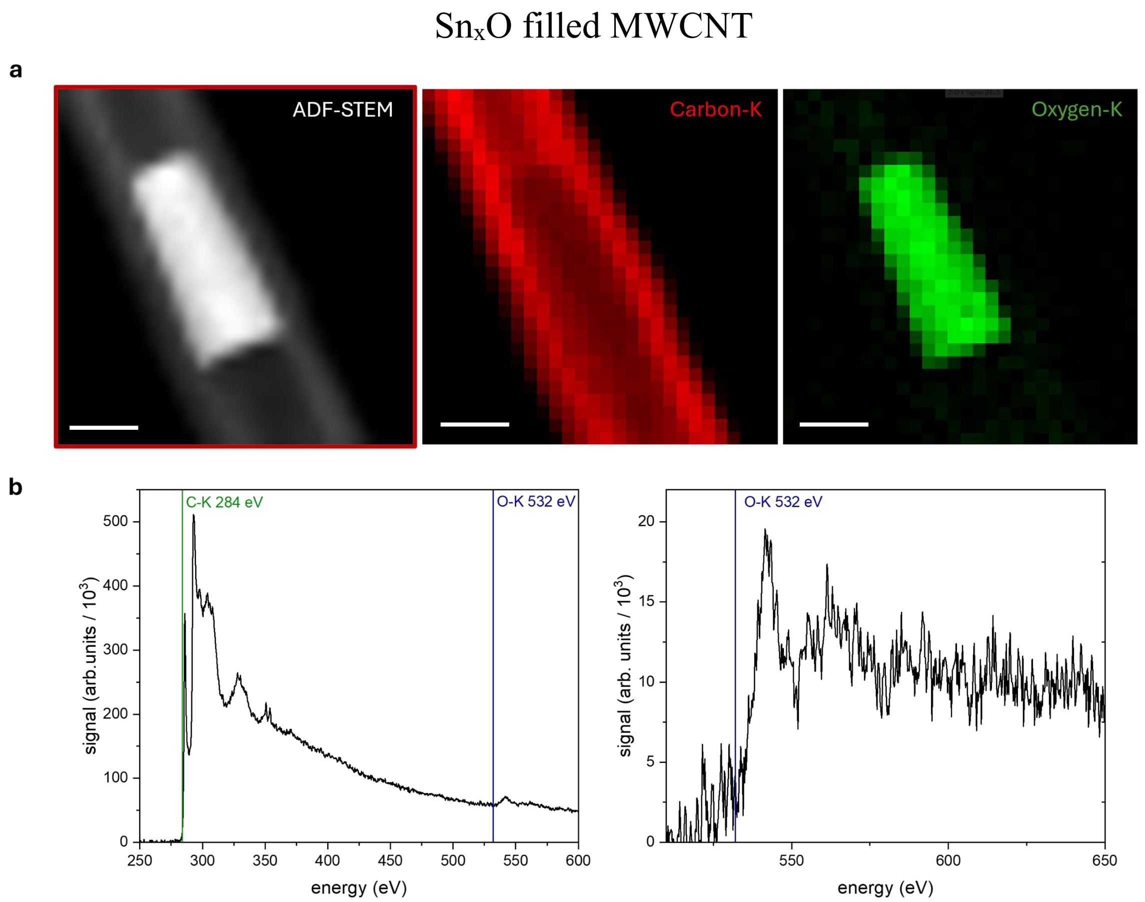

Suppl. Fig. 17: Structural and compositional analysis of Sn_xO nanowire encapsulated within a MWCNT. ***a****, Angular dark-field - scanning transmission electron microscopy (ADF-STEM) micrograph of the Sn_xO nanowire encapsulated within the MWCNT, with corresponding derived elemental maps showing carbon (C-K, red) indicating the MWCNT structure and oxygen (O-K, green) corresponding to the encapsulated region of a Sn_xO nanowire.* ***b****, Electron Energy Loss spectroscopy (EELS) acquired from the same region, showing the high-loss spectrum for the carbon K-edge (284 eV), oxygen K-edge (532 eV), and Sn -M_5 (485 eV) and -M_4 (493 eV) edges, indicating the localised and elemental presence of predominantly carbon and oxygen. Sn M_4/M_5 edges exhibit low scattering cross-sections and partially overlap with the O–K edge.* ***c****, Magnified view of the O-K edge spectra, including the Sn -M_4 and -M_5 edges, illustrating the characteristic near-edge fine structure of the oxygen signal. Scale bar:* ***a-c****, 10 nm.*

Electron Energy Loss Spectroscopy (EELS) spectrum was performed by rastering the electron beam across the sample containing both unfilled MWCNTs and Sn_xO-filled nanowires (as indicated by the annotated red square the ADF-STEM micrograph). The high-loss spectrum reveals a pronounced oxygen-K edge at 532 eV which correlates spatially with the encapsulated Sn_xO nanowire within the MWCNT core.

The spatially localised O–K edge signal is confined to the Sn_xO nanowire and absent from the inner nanotube wall. This stands in contrast to classical condensation models, which predict the formation of continuous thin wetting films via adsorbed liquid layers on confining surfaces prior to condensation. The absence of such a signal suggests that pre-wetting layers do not form under the extreme confinement of the MWCNT core.

The Sn -M_4 (493 eV) and -M_5 (485 eV) edges exhibit weak scattering cross-sections and overlap significantly with the O-K edge at 532 eV, as noted in prior reports[3,4]. However,

the presence of pre-edge features leading up to the oxygen K-edge provides indirect evidence for the presence of Sn, supporting the identification of encapsulated Sn_xO within the nanotube cores. Reference spectra from unfilled regions of the nanotube, as shown in Figure S14, confirm that the observed O-K edge originated primarily from the encapsulation of Sn_xO, rather than residual oxygen from acid oxidation of the MWCNT walls.

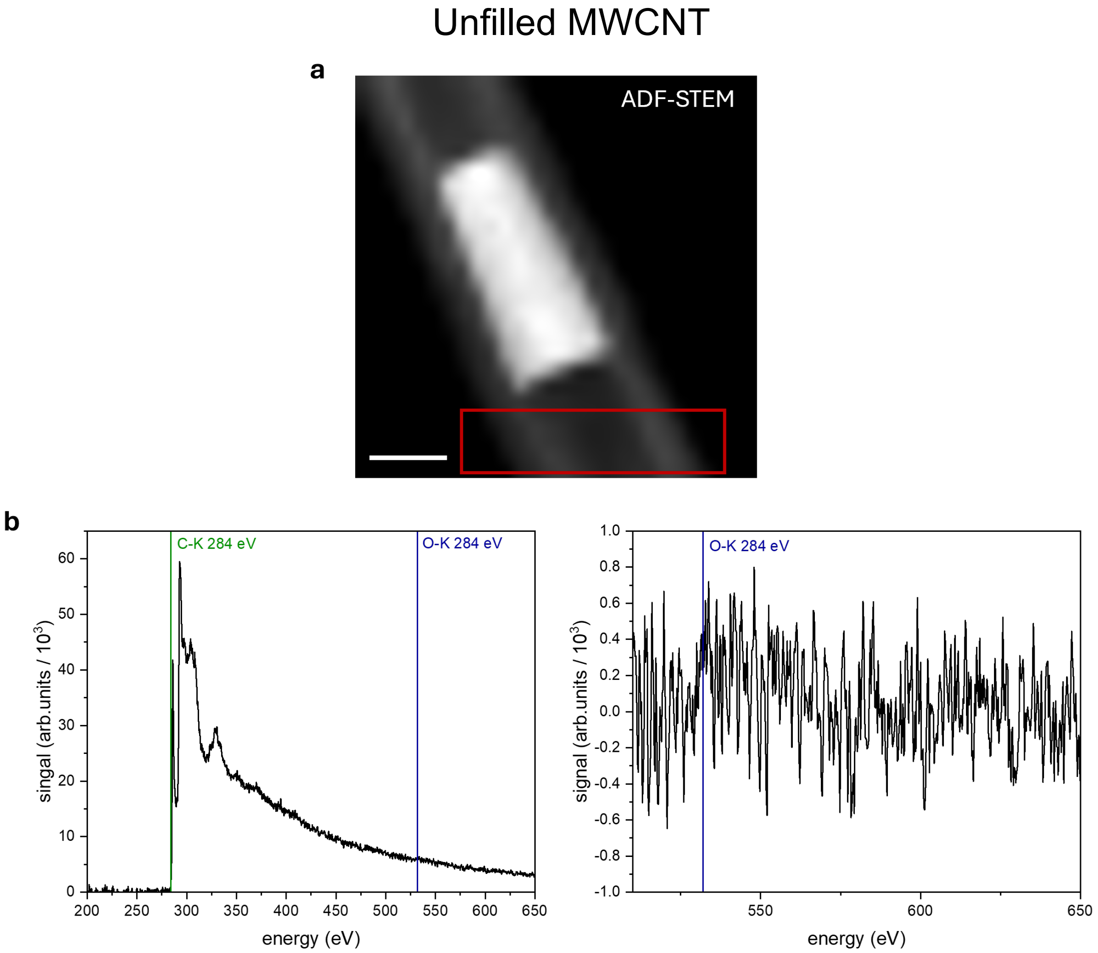

***Suppl. Fig. 18: Electron energy loss spectroscopy (EELS) spectrum comparison of the unfilled oxygen edge. a**, Angular dark-field-scanning transmission electron microscopy (ADF-STEM) micrograph highlights an unfilled section of the acid-treated MWCNT with a red region of interest selected to assess the possible contribution of oxygen from the unfilled MWCNT. **b**, the corresponding EELS spectra show a clear carbon K-edge (284 eV) without a significant oxygen K-edge (532 eV) signal **c**, magnified sub-plot of the O-Kedge with no apparent characteristic near-edge fine structure, indicating that the observed oxygen in filled regions is primarily from the Sn_xO encapsulated nanowire, rather than residual oxygen from the acid treatment of the nanotube. Scale bar: **a**, 10 nm.*

All EELS measurements were acquired as dual spectra to simultaneously capture multiple core-loss edges. The spectra were aligned to the zero-loss peak, and background subtracted using a power-law fit to accurately isolate the core-loss edges.

8. Assessing wettability via contact angle analysis

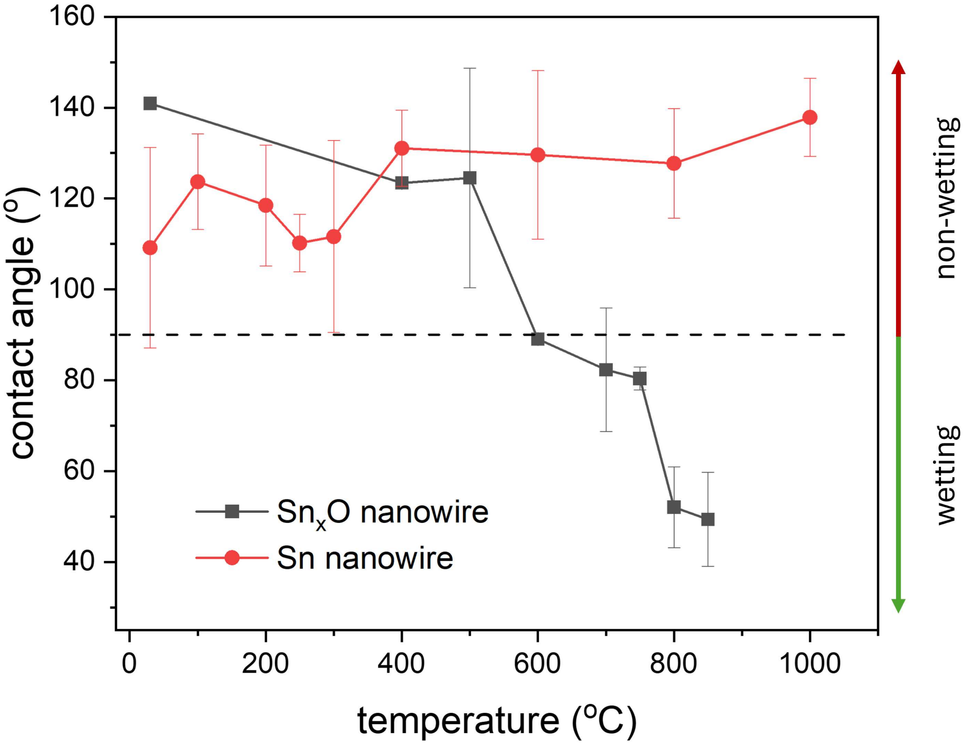

Suppl. Fig. 19: Average contact angle of a Sn_xO and Sn nanowire encapsulated within the MWCNT*. The increase in temperature corresponds to a reduction in the surface tension of the encapsulated, which if wetting to the carbon surface, enables adhesion forces to favorably interact with carbon surface, such as in the case for Sn_xO nanowire. For Sn_xO nanowire, the favorable wetting interactions become more favorable with temperature. Conversely, for Sn nanowire, despite melting at 232 ℃, cohesive forces prevent effective wetting of the carbon surface. The contact angles were measured from multiple nanowire interactions captured through in situ ARTEM data, representing the wetting interaction at the boundary between the carbon surface and the nanowire. Error bars correspond to the standard deviation calculated from the average of multiple contact angle measurements of the Sn_xO and Sn nanowires.*

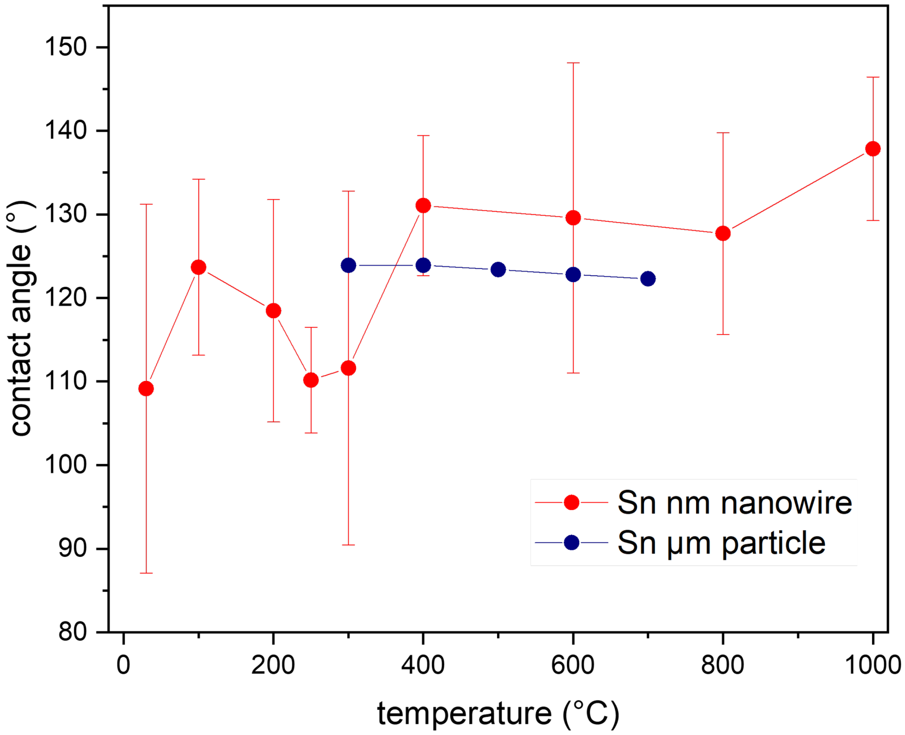

Suppl. Fig. 20: Contact angle comparison between Sn nano- vs. macro- scale wetting behaviour. *Red circles: in situ heating measurements acquired by in situ heating using ARTEM of a Sn nanowire encapsulated in a MWCNT. These nano-wetting data of Sn nanowire exhibit a broad distribution of contact angles (110 – 138°) with large error bars, reflecting the sensitivity of wetting at the nanoscale to local surface effects, such as atomic-scale defects, lattice distortions, and surface curvature. Blue circles: macroscopic sessile-drop data*[5] *extracted from the literature for micrometre scale Sn droplets. The apparent contact angle remains essentially constant at 122 ± 3 ° from room temperature to 800 °C. Error bars correspond to the standard deviation calculated from the average of multiple contact angle measurements of the Sn nanowire.*

9. Filling distribution of Sn_xO nanowires growth over time

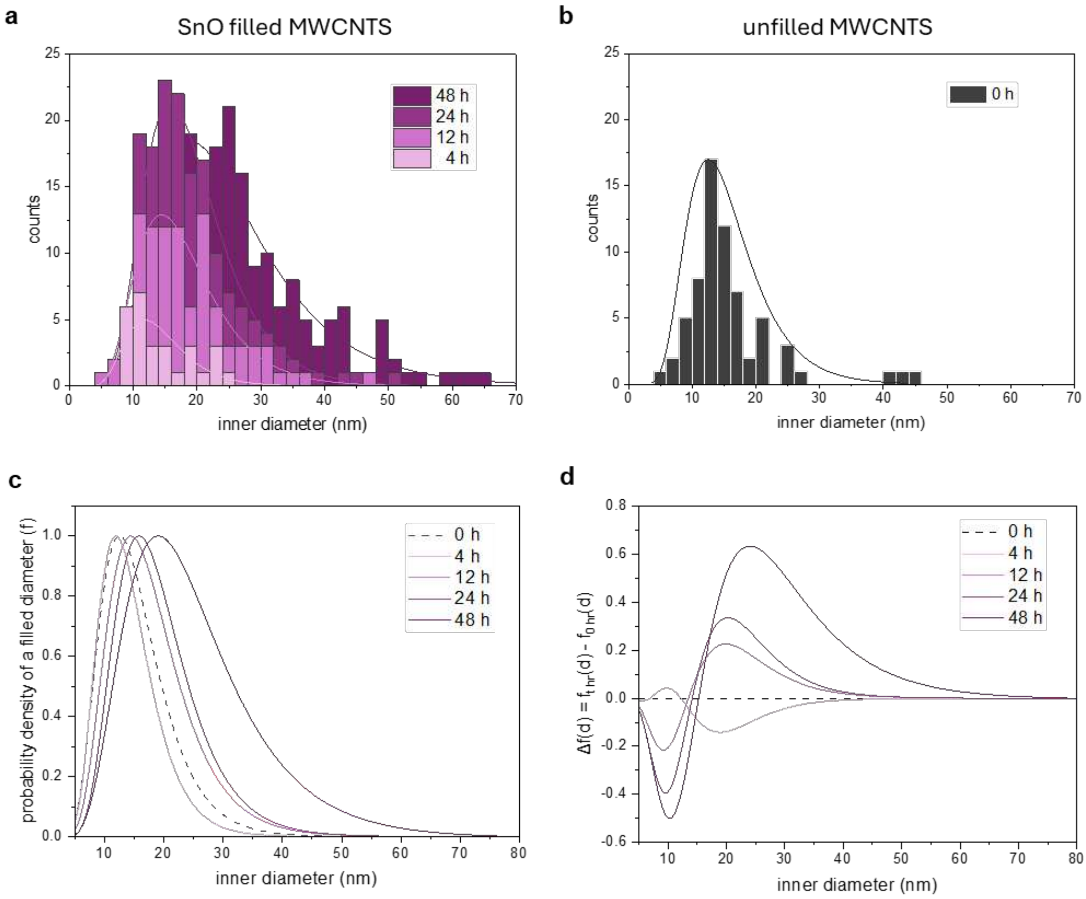

***Suppl. Fig. 21: Nanowire filling dimension distribution within MWCNTs across different annealing times. a**, Histogram showing the inner diameter distribution of Sn_xO-filled MWCNTs surveyed after 4 h, 12 h, 24 h, and 48 h of annealing (left), **b**, alongside the available unfilled MWCNT core distribution at 0 h (right), with a mean inner diameter of 12.55 nm. **c**, Probability density curves for lognormal distributions of filled nanotube diameters at different time points, indicating a progressive increase in mean filled diameters of 11.94 nm for 4 h, 14.47 nm for 12 h, 15.82 nm for 24 h, and 19.06 nm at 48 h. **d**, Differential distribution plot relative to the unfilled 0 h distribution, highlighting a pronounced skew towards larger diameters as annealing progresses. This shift suggests that early-stage filling depletes local vapour pressure, subsequently enabling larger nanotubes to overcome their nucleation barriers as vapour flux accumulates over time. The colour gradient represents vapour annealing time, where light purple corresponds to shorter vapour annealing and darker purple indicates the filling distribution at longer annealing times. Distribution of unfilled available MWCNT diameters is represented a 0 h with a dash line.*

10. Application of the Lucas–Washburn Model to Nanotube Filling Dynamics:

To investigate the growth dynamics of encapsulated Sn_xO nanowires within MWCNTs, we applied the classical Lucas-Washburn (LW) model as a foundational framework for capillary-driven MWCNT filling. The LW model describes the capillary rise of a liquid into a cylindrical core of the tube, balancing the driving capillary pressure, which drives the liquid flow, against the viscous drag, which opposes the advancing meniscus.

The LW equation, derived from the Young-Laplace equation for capillary pressure and Poiseuille's law of viscosity, is given by:

$$L^2 = \frac{\gamma \cos\theta \, d}{4\eta} \cdot t$$

where, L is the length of the nanowire in the tube, γ is the liquid surface tension, θ is the liquid contact angle, η is the viscosity, t is time, and d is filled inner diameter.

The LW model provides a foundational framework for understanding the filling dynamics of Sn_xO within MWCNTs by capturing the balance between capillary driving forces and viscous resistance, which are key factors governing material infiltration in nanoscale capillaries. From our observations, the advancing front of Sn_xO within the MWCNT core is driven by capillary pressure and opposed by viscous drag, resembling the liquid meniscus dynamics described by the LW model. However, direct application of this model is limited due to the vapour-phase nature of Sn_xO condensation within the MWCNT core, discrete nucleation events, and pronounced nanoscale effects influencing viscosity and contact angle. These complexities, coupled with the distribution of available MWCNT diameters, lead to varied filling rates and volumes, necessitating a generalised power-law model to describe the observed diameter dependent volume filling of different sized encapsulated Sn_xO nanowires.

Deriving the power law filling model:

To capture these more complex dynamics, we extend the LW model to a power-law form where the filled volume depends on both the nanotube diameter and the exposed vapour annealing time, resulting in a power-law relation of:

$$V = ad^b$$

where, V is the filled volume (nm^3), d is the diameter (nm), α is the exponent factor reflecting the dimensional scaling ($nm^{0.5}$), including effects from the dynamic contact angle and vapour flux and b is the scaling exponent.

The power law model emerges by combining the diameter dependent filling volume with the cylindrical volume of the tube:

$$V = d^2 L$$

where the volume, V, is equal to the nanotube cross-sectional area (d^2) and the filled length, L (nm). According to the classical LW model, the filling length of a liquid in a tube scale with

the square root of time, reflecting the balance between capillary filling and opposing viscous forces:

$$L = \left(\frac{\gamma \cos\theta \, d}{4\eta} \cdot t\right)^{0.5}$$

For a given temperature series, parameters such as the surface tension, contact angle, and viscosity are assumed to remain constant at constant temperature for each annealing dataset. At a fixed annealing time, the filling length can be approximated to scale as:

$$L \propto d^{0.5}$$

Substituting this length scaling into the volume expression:

$$V \propto d^{2} \cdot d^{0.5} = d^{2.5}$$

Equating to an overall power-law model of:

$$V = \alpha d^{2.5}$$

where the prefactor α captures the combined effects of surface tension, contact angle, viscosity, and temperature, as well as the dynamic interactions specific to the confined size of the MWCNT core.

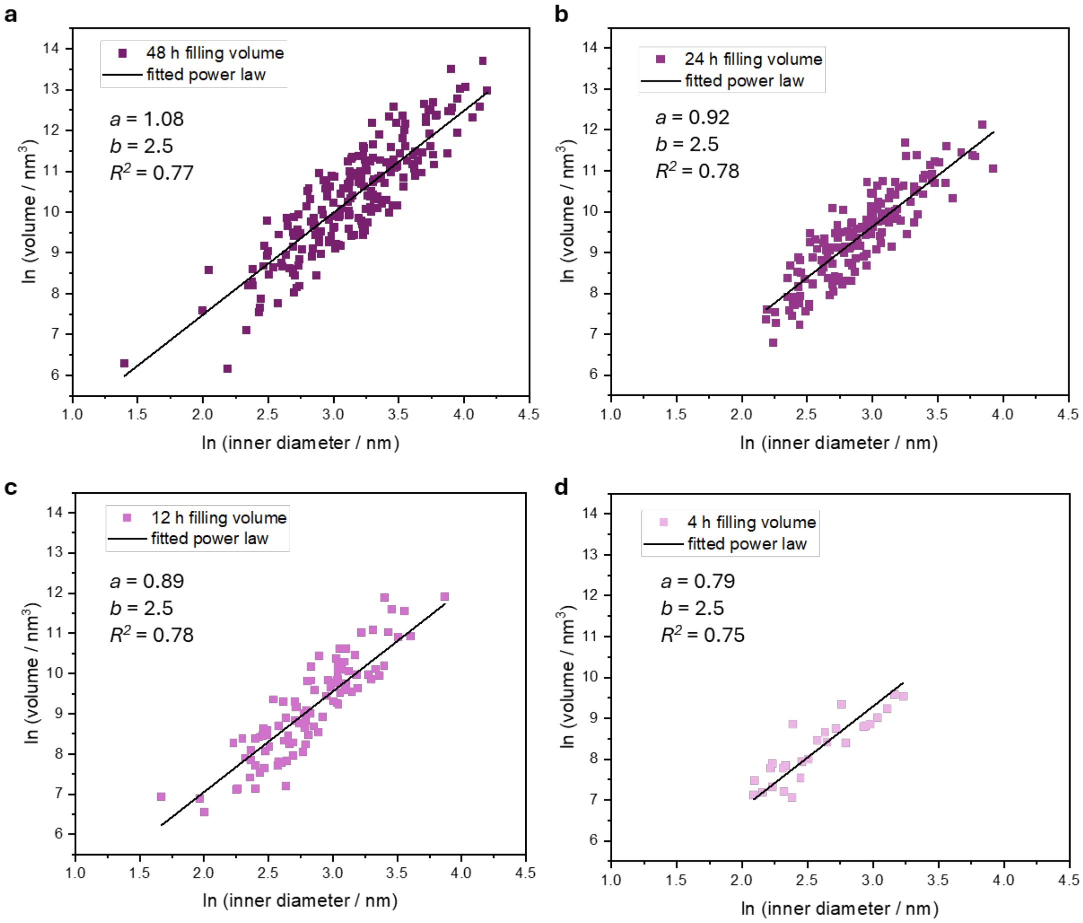

Suppl. Fig. 22: Modelling the Encapsulated Nanowire Volume. *Linearised power-law scaling for* Sn_xO *nanowire filling within MWCNTs at different annealing times.* ***a****, 48 h,* ***b****, 24 h,* ***c****, 12 h, and* ***d****, 4 h. Each plot shows the natural logarithm of filled volume versus the natural logarithm of inner diameter, with fitted power-law exponents consistently around b = 2.5, reflecting the combined effects of capillary-driven condensation and vapour-phase flux within the confined nanotube structure. The colour gradient, ranging from light to dark purple, corresponds to increasing vapour annealing time, with light purple indicating shorter annealing durations and dark purple representing longer vapour annealing time.*

The colour gradient represents vapour annealing time, where light purple corresponds to shorter vapour annealing and darker purple indicates the filling distribution at longer annealing times.

The fitted power-law model ($V = ad^{2.5}$) was applied across different annealing times to analyse how the filling mechanism evolves. The extracted prefactor, a, and the R^2 coefficient values are summarised for each time series in Table S6.

Table S6: Fitted power-law parameters for each annealing time.

**Annealing time (h)**	**prefactor α (nm$^{0.5}$) 0**	**R^2 coefficient**
**4**	0.79	0.75
**12**	0.89	0.79
**24**	0.92	0.78

**48**	1.08	0.77

The gradual increase in the prefactor, a, from 0.79 at 4 h to 1.08 at 48 h indicates a progressive enhancement in overall filling efficiency for larger diameter nanotubes with greater annealing times.

In the early stages of filling, smaller-diameter nanotubes, which have lower nucleation barriers due to their higher curvature and reduced Kelvin radii, reach their condensation thresholds first. This initial nucleation locally depletes the surrounding vapour, effectively reducing the vapour pressure near these smaller tubes. As a result, the relative saturation near larger-diameter tubes increases with time to facilitate nanowire nucleation and growth. Consequently, the higher filling efficiency of larger nanotubes, which benefit from greater vapour flux and reduced viscous resistance, progressively enhances the collective growth rate, leading to larger nanowire volumes over time. The increasing filling rates of larger nanotubes over time are reflected in the rising prefactor, α, in each fitted power-law model, capturing the combined effects of enhanced vapour flux and reduced internal resistance, which together drive the efficient filling of larger-diameter nanotubes. The increased filling of larger-diameter nanotubes, which only become activated at longer annealing times due to their higher nucleation thresholds, indicates that the overall kinetic filling process is not limited by vapour flux.

The growth of nanowires within nanotubes is opposed by internal viscous drag, which becomes increasingly significant at the nanoscale due to the greater surface-to-volume interactions and confinement of the nanotube core. As the liquid meniscus advances within the confined nanotube core, this viscous resistance typically slows the filling rate, consistent with the classical Lucas-Washburn model and the observed b = 2.5 power-law scaling for volume filling over time. However, this drag is partially offset by the simultaneous rise in local vapour saturation, driven by the progressive depletion of smaller filled tubes. As the local vapour pressure increases, it sustains a net positive driving force for capillary flow within the MWCNT core, counteracting the increasing internal viscous drag that typically resists nanowire growth and promoting the formation of continuously encapsulated nanowires.

The moderately high R^2 values (0.75 to 0.80) observed across all time points indicate that the power-law model with a fixed exponent of b = 2.5 provides a reasonably good fit to the observed filling data, capturing the dominant trend of nanowire growth within the confined MWCNT structure.

For comparison, each annealing time series was fitted with the power-law ($V = \alpha d^b$), which allows both the prefactor (α) and the scaling exponent (b) to vary independently in Figure S12 and summarised in Table S7.

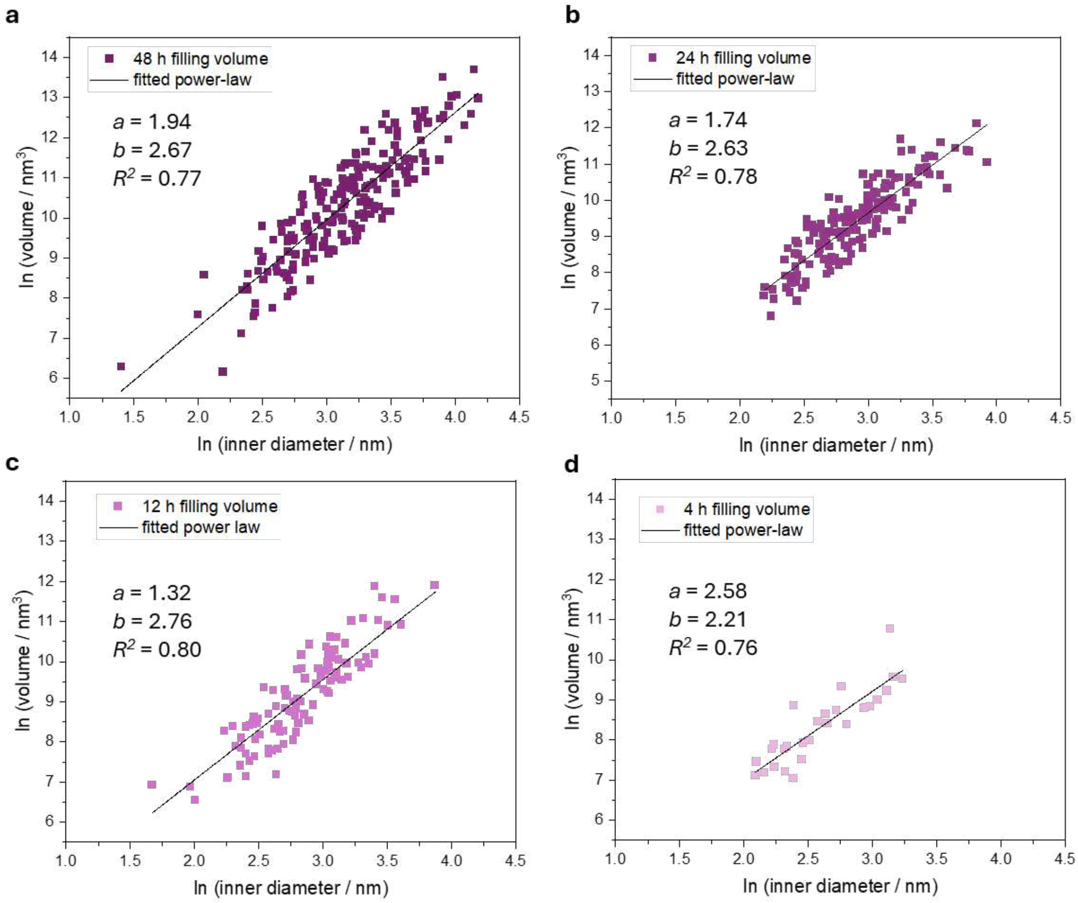

Suppl. Fig. 23: Modelling the volume of filling with unfixed fitting parameters. *Linearised power-law model to fit for both the prefactor (α) and exponent (b) for the volume of Sn_xO-nanowire filled confined with MWCNTs with an unconstrained power-law fitting.* ***a****, 48 h,* ***b****, 24 h,* ***c****, 12 h,* ***d****, 4 h. Each plot is fitted with the nature natural logarithm of filled volume (V) versus the natural logarithm inner diameter (d) fitted with a liner regression. The colour gradient from light to dark purple indicates increasing vapour annealing time, from early to longer vapour annealing times.*

Table S7: Fitting Parameters for Non-Fixed Power-Law Model

**Annealing time (h)**	**α ($nm^{0.5}$)**	**b**	**R^2 coefficient**
**4**	2.58	2.21	0.76
**12**	1.32	2.76	0.80
**24**	1.74	2.63	0.78
**48**	1.94	2.67	0.77

11. Box plot statistic of growth dynamics

Suppl. Table 8: Summary statistics for Sn_xO-filled nanotube diameter, nanowire length, and aspect ratio with increasing annealing time. (All values displayed in nm units).

filled inner diameters

annealing time (h)	count	mean	std	min	25 %	50 %	75 %	max	IQR
4	28	14.35	5.27	8.08	10.16	12.70	18.82	25.31	8.66
12	97	18.05	7.22	5.31	13.16	16.41	21.31	47.95	8.16
24	163	19.30	7.68	8.92	13.94	17.40	22.83	50.76	8.90
48	213	25.40	11.28	4.05	17.34	23.24	30.91	65.31	13.58

nanowire length

annealing time (h)	count	mean	std	min	25 %	50 %	75 %	max	IQR
4	28	30.10	18.50	11.51	21.46	24.33	31.90	10.44	10.44
12	97	45.23	30.18	8.02	25.41	37.36	55.60	187.73	30.18
24	163	49.13	29.43	11.73	26.20	44.20	62.56	209.58	36.36
48	213	85.75	61.62	6.94	40.57	70.16	115.14	348.49	74.60

aspect ratio

annealing time (h)	count	mean	std	min	25 %	50 %	75 %	max	IQR
4	28	2.28	1.28	0.99	1.17	2.06	2.72	6.35	1.54
12	97	2.60	1.50	0.58	1.48	2.25	3.23	8.02	1.76
24	163	2.68	1.50	0.56	1.60	2.27	3.19	8.54	1.59
48	213	3.61	2.45	0.77	1.73	2.89	4.65	13.43	2.92

12. References:

1. Mäki-Jaskari, M. A. & Rantala, T. T. Possible structures of nonstoichiometric tin oxide: the composition Sn 2 O 3. *Model Simul Mat Sci Eng* 12, 33–41 (2004).

2. White, T. A., Moreno, M. S. & Midgley, P. A. Structure determination of the intermediate tin oxide Sn 3 O 4 by precession electron diffraction. *Zeitschrift für Kristallographie* 225, 56–66 (2010).

3. Moreno, M. S., Egerton, R. F. & Midgley, P. A. Differentiation of tin oxides using electron energy-loss spectroscopy. *Phys Rev B* 69, 233304 (2004).

4. Moreno, M. S., Egerton, R. F., Rehr, J. J. & Midgley, P. A. Electronic structure of tin oxides by electron energy loss spectroscopy and real-space multiple scattering calculations. *Phys Rev B* 71, 035103 (2005).

5. Yuan, Z. F. *et al.* Surface Tension and Its Temperature Coefficient of Molten Tin Determined with the Sessile Drop Method at Different Oxygen Partial Pressures. *J Colloid Interface Sci* 254, 338–345 (2002).
