# Supplementary material for: Capturing atomic wetting dynamics in real time
Source: Nat Commun. 2025 Nov 28;16:11350. doi: 10.1038/s41467-025-66416-1 (PMC12727699; doi:10.1038/s41467-025-66416-1)
Supplement: Supplementary file 2 — Transparent Peer Review file [file 41467_2025_66416_MOESM2_ESM.pdf]

nature portfolio

Peer Review File

Capturing atomic wetting dynamics in real time

Corresponding Author: Professor Nicole Grobert

This file contains all reviewer reports in order by version, followed by all author rebuttals in order by version.

Version 0:

Reviewer comments:

Reviewer #1

(Remarks to the Author)
In this manuscript authors investigated the Sn nanowire growth mechanism using in situ atomic-resolution transmission electron microscopy (ARTEM) combined with a deep learning convolutional neural network (CNN) capable of differentiating liquid and solid SnO, as well as intermediate SnO_x phases at the atomic scale. It is indicated the crucial role of a wetting interface between the liquid precursor and the nanotube wall, that the traditional models, such as Kelvin and Lucas–Washburn, usually fail to capture.These findings establish a predictive framework for nanowire growth under confinement, and provide a deep understanding and scalable approach to the synthesis of advanced nanomaterials. This manuscript was well organized and the conclusions can be supported by the data.It can be accepted after some minor revisions.
1.In SI part, Fig.S3（b）,the EDS line profiles across the SnO-filled nanotubes, the C line profile is very strange and it does not seem like the core-shell structure of carbon nanotubes. It should be a saddle-type profile. Why? Please check and repeat it.

(Remarks on code availability)

Reviewer #2

(Remarks to the Author)
Manuscript ID: NCOMMS-25-48573-T

Manuscript Title: Capturing atomic wetting dynamics in real time

Comments to the Author:

This manuscript proposes a two-stage mechanism for the formation of metallic nanowires within multi-wall carbon nanotubes (MWCNTs), attributing their growth to atomic wetting effects. This study utilized in-situ atomic-resolution transmission electron microscopy (ARTEM) combined with deep learning to track the evolution of SnxO at the atomic scale, to understand the underlying mechanism. The paper is well-written and effectively communicates the research scope, presenting a predictive framework for confined nanowire growth. This framework is beneficial for future scalable, bottom-up fabrication of nanoscale devices. Below are my specific comments:

Main suggestions:

1. The manuscript is confusing due to the inconsistent use of terms, particularly "SnO" and "SnxO," when describing the components/phases within the MWCNT. I believe "SnxO" is a more accurate term to describe the actual mixed phases of oxides within the MWCNT at the initial status of the samples, whereas "SnO" should only be used to refer to the SnO solid precursor. This aligns with the definition of class labels used in CNN classification, such as amorphous SnxO, liquid SnxO, intermediate Sn3O4, and Sn2O3 phases. For example, abstract (P1, L20-21), "...convolutional neural network (CNN) capable of differentiating liquid and solid SnO, as well as intermediate SnO_x phases…", it is suggested to use "amorphous SnxO, liquid SnxO, intermediate Sn3O4, and Sn2O3 phases" instead. Similarly, please carefully check captions for Figure 3(P7, L202), Extended Data Figure 3 (P20, L690), Figure S4 and S5 (Sn3O4 should be SnxO) for consistency.

2. The study's in-situ experiment did not directly visualize nanowire filling/growth. While the observation of vaporization aids understanding of wetting and droplet formation mechanisms, it should not be described as an in-situ observation of nanowire growth. For example, in the abstract, P1 L25-26, “time-dependent filling” refers to in-situ observation of SnO/Sn nanowire vaporization, not direct growth. Please ensure consistency throughout the manuscript to prevent any potential confusion for the audience.

3. The Extended Data Figure 4 shows the Location analysis of SnO nanowires to the tube opening. While it can be inferred that through these images, nanowire growth initiated at the tube openings, does it explain the mechanism of nanowire growth via confined condensation and wetting as presented in Figure 4 on P9? Please clarify this.

4. P4, L131 – “Figure 2 presents a CNN-labelled in situ heating series, where the model distinguishes key pixel classes involved in the encapsulation process: the MWCNT wall (dark blue), the solid phase of the encapsulated amorphous SnxO nanowire (green), the liquid-phase SnxO (yellow), small intermediate crystalline oxides, (Sn3O4, dark blue, and Sn2O3, dark red) formed by disproportionation, and the empty MWCNT core (light blue)” and P5, Figure 2.

At the atomic level, both liquid and amorphous structures lack the long-range order that is characteristic of crystalline solids. One might expect the high-resolution TEM images to have a blurry, disordered appearance, lacking distinct lattice fringes or patterns that characterize crystalline material phases. While elemental characterization for MWCNTs through EDS has been presented in Figure S3 in the SI, and characterization of lattice structures for Sn2O3 and Sn3O4 as reaction intermediates has been demonstrated in Figures S6 and S7 in the SI, a detailed description of how amorphous and liquid SnxO were distinguished should be presented or commented on. While the authors attribute the observation of liquid SnxO phase during meniscus formation on P6, L165, “Heating the SnO nanowire to 750 °C produces a curved meniscus against the CNT wall (Figure 2g–166 j), indicative of a condensed liquid phase (yellow)” was this the only indicator used to distinguish the two during image analysis? Please clarify.

5. For the temperature-dependent wetting behavior of SnxO nanowires, what are the speeds of the evolution steps? Which transition is most sensitive to incremental temperature changes? What is the frame rate of the in-situ imaging, and is it fast enough to capture these transitions? The observation of Sn3O4 appearing and disappearing in some figures (e.g., Fig 2, navy blue) requires interpretation.

6. The CNN training dataset (P4, L123, and P15, L518) involved manually labeling 1,000,000 image patches. This is a very human-intensive task, especially given the quantity and difficulty of distinguishing subtle lattice fringes/patterns. Please clarify if any image processing techniques (e.g., FFT filtering, denoising, contrast enhancement) were applied. Additionally, what are the classification accuracies for each class? Was there any class imbalance in the training dataset that might have affected detection accuracy? It is suggested to provide more details on the challenges and limitations of the developed model, considering the characteristics of these datasets.

7. In SI, Fig. S5 (r-s), were there mixed phases that occurred, as evidenced by the formation of regions in purple? Did you observe any beam damage or electron-dose sensitivity behavior for some phases/components?

Minor suggestions:

1. P2, L68 – remove “recent”; else if there is additional text after, please add the same.

2. P2, L85 – “Analysis of both the cubic and tetragonal phases of β-Sn is shown in Extended Data Figures.” Are the authors referring to the cubic β-Sn metastable phase and the tetragonal β-Sn stable phase here?

3. Several figure numbering issues. P6, L152, “Figure 2d-u” should be “Figure 2b-u”, L159, “Figure 2d” should be “Figure 2b”, L162, “Figure S4c-q” should be “Extended Data Figure 2”. P20 L689, “figure 2 d-w” should be “ figure 2 b-u”. All figures in the SI, section 3.

4. P9, L275, replace “time” with “vapor annealing time” for clarification, as these are not in-situ real-time observations, but rather static observations of samples prepared using different vapor annealing times.

5. In the SI, section 6 EELS analysis, P18, Figure S9's caption, “Showing the low-loss spectrum for the carbon K-edge(284 eV) and the high-loss spectrum for the oxygen K-edge(532 eV)”, remove” low-loss spectrum” and “high-loss spectrum” as both C-K and O-K edges are core-loss features. Figure S14 for Sn M4,5 edges was missing.

6. In the SI, Fig. S7 is missing scale bar information.

7. In the SI, P8 – “ARTEM micrographs exhibit significant contrast variance, as the micrograph intensity depends on the interference of electron waves that pass through and exit the sample. This phase contrast is highly sensitive to experimental conditions such as defocus, sample thickness, and beam brightness, leading to variations in the appearance of atomic features. This contrast is highly sensitive to experimental TEM parameters, including defocus, beam brightness, and sample thickness, all of which can alter the appearance of atomic-scale features (either as bright or dark contrast).”
The last two sentences are repeated.

8. In the SI, P18 – “The localized distribution of the oxygen-K edge signal, contrasts with the continuous wetting layer that would be expected if classical vapour condensation mechanisms.”
It seems that there is some text missing at the end of this sentence.

9. In the SI, P20 – “The LW model describes the capillary rise of a liquid into a cylindrical core of the tube, balancing the driving capillary pressure against, which drives the liquid flow, against the viscous drag, opposed the advancing meniscus.” Against (the first instance in the above sentence should be removed). Additionally, should “opposed” be changed to “which opposes”?

10. In the SI, P21 – Figure S11
The power law prefactor in Figure S11 (b) should be 0.92 instead of 0.0.92
Figure S11(c) and (b) have the same caption. Should (c) be 12 hr annealing time? If yes, please modify the caption on Page 22.

11. In the SI, P23 – Figure S12 and Table S7
The fit parameters for Non-Fixed Power-Law Model in Figure S12 (a)-(d) and Table S7 do not match. If both prefactor and power law scaling exponent were floated, how likely is it that the pre-factors in Table S7 and S6 match ? Please check.

12. Can the authors discuss the effects of MWCNT surface chemistry/defects on metal nanowire wetting? Additionally, experimental techniques such as in-situ 4D STEM and atomic-scale simulations (DFT, AIMD, etc) could further elucidate the fundamental mechanism of this phenomenon. Expanding the study to include these approaches in the future would be beneficial.

(Remarks on code availability)

Reviewer #3

(Remarks to the Author)
I co-reviewed this manuscript with one of the reviewers who provided the listed reports. This is part of the Nature Communications initiative to facilitate training in peer review and to provide appropriate recognition for Early Career Researchers who co-review manuscripts.

(Remarks on code availability)

Version 1:

Reviewer comments:

Reviewer #1

(Remarks to the Author)
This manuscript was revised according to the comments of referees. Now I am pleased to recommend the acceptance in the current form.

(Remarks on code availability)

Reviewer #2

(Remarks to the Author)
Thank you for addressing the comments and revising the manuscript. The manuscript in its current form is suitable for publication.

(Remarks on code availability)

Reviewer #3

(Remarks to the Author)
I co-reviewed this manuscript with one of the reviewers who provided the listed reports. This is part of the Nature Communications initiative to facilitate training in peer review and to provide appropriate recognition for Early Career Researchers who co-review manuscripts.

(Remarks on code availability)

Open Access This Peer Review File is licensed under a Creative Commons Attribution 4.0 International License, which permits use, sharing, adaptation, distribution and reproduction in any medium or format, as long as you give appropriate credit to the original author(s) and the source, provide a link to the Creative Commons license, and indicate if changes were made.
In cases where reviewers are anonymous, credit should be given to 'Anonymous Referee' and the source.
The images or other third party material in this Peer Review File are included in the article's Creative Commons license, unless indicated otherwise in a credit line to the material. If material is not included in the article's Creative Commons license and your intended use is not permitted by statutory regulation or exceeds the permitted use, you will need to obtain permission directly from the copyright holder.
To view a copy of this license, visit https://creativecommons.org/licenses/by/4.0/

Peer Review Comments and Replies:

"Capturing atomic wetting dynamics in real time".

We thank the referees for their time, feedback, and supportive comments, which have constructively contributed to improving and refining our manuscript. Each comment (*black italics*) has been carefully addressed, and our detailed responses are provided below in blue text.

In addition to the specific revisions prompted by the reviewers' comments, we have made a small number of additional edits inspired by their feedback. These consist primarily of minor typographical corrections and wording improvements to enhance clarity and readability.

All changes have been tracked in both the main manuscript and the Supplementary Information for clarity.

***Reviewer #1** (Remarks to the Author):*

In this manuscript authors investigated the Sn nanowire growth mechanism using in situ atomic-resolution transmission electron microscopy (ARTEM) combined with a deep learning convolutional neural network (CNN) capable of differentiating liquid and solid SnO, as well as intermediate SnO_x phases at the atomic scale. It is indicated the crucial role of a wetting interface between the liquid precursor and the nanotube wall, that the traditional models, such as Kelvin and Lucas–Washburn, usually fail to capture. These findings establish a predictive framework for nanowire growth under confinement and provide a deep understanding and scalable approach to the synthesis of advanced nanomaterials.

This manuscript was well organized, and the conclusions can be supported by the data. It can be accepted after some minor revisions.

1.In SI part, Fig.S3 （b）, the EDS line profiles across the SnO-filled nanotubes, the C line profile is very strange, and it does not seem like the core-shell structure of carbon nanotubes. It should be a saddle-type profile. Why? Please check and repeat it.

Upon re-examination of the data and acquisition of additional EDS line profiles, we were able to obtain a representative dataset that exhibits the expected saddle-type (M-shaped) carbon signal profile across the nanotube. These examples of the EDS signal more clearly reflects the core–shell structure of the MWCNT and confirms that the highest carbon intensity corresponds to the nanotube walls.

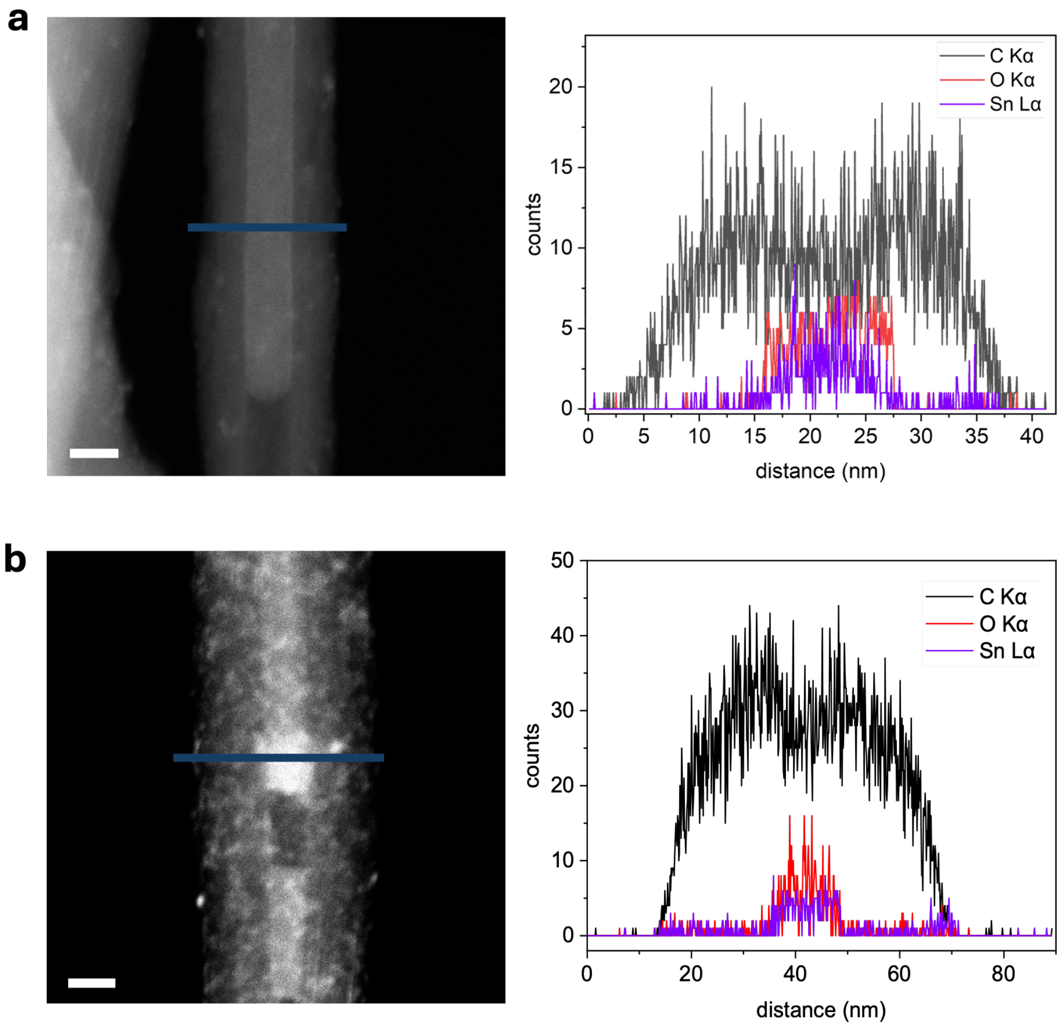

*Figure R1: Updated EDX line scan profiles acquired from two different Sn_xO@MWCNT samples (**a** and **b**), showing the location of the line scans on corresponding ADF-STEM micrographs, along with the associated EDX line profiles of C, Sn, and O. The C signal exhibits the expected saddle-shaped profile, consistent with the core-shell structure of multiwalled carbon nanotubes, while Sn and O signals remain localized within the inner channel, confirming encapsulation. Scale bar: **a**, 10 nm; **b**, 20 nm.*

We have now included this updated line profile in the revised Supplementary Figure S3. Importantly, the Sn and O signals remain consistently localised within the interior of the tube, further supporting our conclusion of Sn_xO confinement inside the MWCNT core.

Reviewer #2 *(Remarks to the Author):*

Manuscript ID: NCOMMS-25-48573-T

Manuscript Title: Capturing atomic wetting dynamics in real time

Comments to the Author:

This manuscript proposes a two-stage mechanism for the formation of metallic nanowires within multi-wall carbon nanotubes (MWCNTs), attributing their growth to atomic wetting effects. This study utilized in-situ atomic-resolution transmission electron microscopy (ARTEM) combined with deep learning to track the evolution of SnxO at the atomic scale, to understand the underlying mechanism. The paper is well-written and effectively communicates the research scope, presenting a predictive framework for confined nanowire growth. This framework is beneficial for future scalable, bottom-up fabrication of nanoscale devices. Below are my specific comments:

Main suggestions:

1. *The manuscript is confusing due to the inconsistent use of terms, particularly "SnO" and "SnxO," when describing the components/phases within the MWCNT. I believe "SnxO" is a more accurate term to describe the actual mixed phases of oxides within the MWCNT at the initial status of the samples, whereas "SnO" should only be used to refer to the SnO solid precursor. This aligns with the definition of class labels used in CNN classification, such as amorphous SnxO, liquid SnxO, intermediate Sn3O4, and Sn2O3 phases. For example, abstract (P1, L20-21), "…convolutional neural network (CNN) capable of differentiating liquid and solid SnO, as well as intermediate* SnO_x *phases…", it is suggested to use "amorphous SnxO, liquid SnxO, intermediate Sn3O4, and Sn2O3 phases" instead. Similarly, please carefully check captions for Figure 3(P7, L202), Extended Data Figure 3 (P20, L690), Figure S4 and S5 (Sn3O4 should be SnxO) for consistency.*

We agree with the suggestion and have adopted Sn_xO as the most accurate and consistent representation of the mixed phases oxides encapsulated nanowires throughout the manuscript, given that SnO precursor disproportionates into a mixed oxide species.

2. *The study's in-situ experiment did not directly visualize nanowire filling/growth. While the observation of vaporization aids understanding of wetting and droplet formation mechanisms, it should not be described as an in-situ observation of nanowire growth. For example, in the abstract, P1 L25-26, "time-dependent filling" refers to in-situ observation of SnO/Sn nanowire vaporization, not direct growth. Please ensure consistency throughout the manuscript to prevent any potential confusion for the audience.*

The following sentence have been amended to clarify and describe the observation of phase transformation which enables inference into the encapsulation mechanism.

Original P1, L25-26: ‘Instead, these confined systems are dominated by nucleation kinetics, vapour flux, and interfacial condensation, which collectively drive the time-dependent filling observed in our ARTEM experiments.’

Revised P1, L25-26: ‘Instead, these confined systems are dominated by nucleation kinetics, vapour flux, and interfacial condensation, which collectively drive the time-dependent nanowire growth within the CNT core.’

Original P1, L18 – 21: Using in situ atomic-resolution transmission electron microscopy (ARTEM) combined with a deep learning convolutional neural network (CNN) capable of differentiating liquid and solid Sn_xO, as well as intermediate Sn_xO phases, we track nanowire growth at the atomic scale.

Revised (P1, L18 – 22): Using in situ atomic-resolution transmission electron microscopy (ARTEM) and a deep learning convolutional neural network (CNN) capable of classifying liquid, solid, and intermediate Sn_xO phases, we uncover the nanowire formation mechanism by tracking the cascade of thermally induced phase transitions (from solid to liquid to vapour) directly at atomic resolution.

Original P1, L24-26: Instead, these confined systems are dominated by nucleation kinetics, vapour flux, and interfacial condensation, which collectively drive the time-dependent filling observed in our ARTEM experiments.

Revised (P1, L25-27): Instead, these confined systems are dominated by nucleation kinetics, vapour flux, and interfacial condensation, which collectively drive the time-dependent filling that is statistically observed.

Original P4, L108-110: To capture the high-spatial-resolution vapour condensation dynamics that drive nanowire formation directly within the MWCNT core, we imaged the mechanism in real time under high vacuum and high temperature conditions within a transmission electron microscope (TEM).

Revised (P4, L109-114): To capture the thermally induced solid–liquid–vapour transitions relevant to nanowire formation, we imaged the dynamic phase evolution of Sn_xO in real-time under high-vacuum, high-temperature conditions within a transmission electron microscope (TEM). While these observations reflect the reverse sequence of nanowire growth, providing direct insight into the wetting interface behaviour, interfacial condensation, and the liquid-phase dynamics that govern confined nanowire formation within MWCNTs.

Original P6, L152-154: Applied frame-by-frame to the in situ ARTEM series (Figure 2d–u), the CNN tracks the nanowire through three distinct stages: green amorphous solid, a yellow liquid that spreads along the CNT wall, and a light-blue vapour that leaves the core vacant.

Revised (P6, L156-159): Applied frame-by-frame to the in situ ARTEM series (Figure 2b–u), the CNN tracks the evolution of the nanowire through three distinct thermally induced phase states: green amorphous solid, a yellow liquid phase along the CNT wall, and light blue representing empty nanotube core as liquid Sn_xO enters the vapour phase leaving it vacant.

Original P6, L154-158: The sequence reveals a series of non-equilibrium, interface-driven events, including melting, wetting, and vaporisation: upon sample heating, the encapsulated nanowire transitions from solid to liquid, accompanied by enhanced wetting of the nanotube wall, followed by gradual vaporisation reflected in the continuous nanowire shrinkage.

Revised (P6, L159-162): The sequence reveals a series of non-equilibrium, interface-driven events following melting, wetting, and vaporisation. Upon heating, the encapsulated nanowire transitions from solid to liquid, accompanied by wetting of the nanotube wall, followed by gradual vaporisation, evident as continuous shrinkage of the nanowire.

Original P6, L172-173: Such confinement is supported by the initiation of nanowire growth at nanotube openings (Extended Data Figure 4), where condensation is favoured.

Revised (P6, L176-180): ADF-STEM images (Extended Data Figure 4) reveal Sn_xO nanowires consistently positioned just inside the open-ended nanotube. This spatial localisation of Sn_xO nanowires supports a Kelvin-driven condensation mechanism: as Sn_xO vapour enters the confined nanotube, the reduced equilibrium vapour pressure favours localised condensation into a liquid droplet, which subsequently elongates inward under capillary forces and solidifies into a nanowire.

3. *The Extended Data Figure 4 shows the Location analysis of SnO nanowires to the tube opening. While it can be inferred that through these images, nanowire growth initiated at the tube openings, does it explain the mechanism of nanowire growth via confined condensation and wetting as presented in Figure 4 on P9? Please clarify this.*

Extended Data Figure 4 presents ADF-STEM images showing multiple Sn_xO nanowires located within a few nm of carbon nanotube opening. While these static ADF micrographs do not capture the dynamic growth process directly, they support the proposed

mechanism in Figure 4 by indicating a spatially consistent nucleation near the nanotube openings. The location of the Sn_xO nanowires aligns with the confined condensation model governed by the Kelvin effect, whereby the entry of Sn_xO vapour into the nanotube results in a local drop in equilibrium vapour pressure, inducing condensation of Sn_xO vapor into a liquid droplet. The localisation of the nanowires just within the entrance implies that the confined geometry promotes condensation upon vapour ingress. Following condensation, the liquid phase is stabilised and elongates slightly inward under capillary forces, before solidifying as the system cools. Thus, the ADF-STEM images reinforce the proposed growth mechanism: entry of Sn_xO vapor, confined condensation (Kelvin effect) which leads to the formation of liquid Sn_xO nanowire droplet, leading to capillary driven growth of the nanowire within the core of the nanotube.

4. *P4, L131 – "Figure 2 presents a CNN-labelled in situ heating series, where the model distinguishes key pixel classes involved in the encapsulation process: the MWCNT wall (dark blue), the solid phase of the encapsulated amorphous SnxO nanowire (green), the liquid-phase SnxO (yellow), small intermediate crystalline oxides, (Sn3O4, dark blue, and Sn2O3, dark red) formed by disproportionation, and the empty MWCNT core (light blue)" and P5, Figure 2.*

At the atomic level, both liquid and amorphous structures lack the long-range order that is characteristic of crystalline solids. One might expect the high-resolution TEM images to have a blurry, disordered appearance, lacking distinct lattice fringes or patterns that characterize crystalline material phases. a detailed description of how amorphous and liquid Sn_xO *were distinguished should be presented or commented on.*

While the authors attribute the observation of liquid SnxO phase during meniscus formation on P6, L165, "Heating the SnO nanowire to 750 °C produces a curved meniscus against the CNT wall (Figure 2g–166 j), indicative of a condensed liquid phase (yellow)" was this the only indicator used to distinguish the two during image analysis? Please clarify.

CNN differentiation between amorphous and liquid Sn_xO phases

The CNN distinguishes between amorphous and liquid Sn_xO based on subtle differences in local intensity and texture. Amorphous Sn_xO regions exhibit higher intensity gradients, more heterogeneous contrast, and coarser granularity, reflecting their disordered internal structure. In contrast, liquid Sn_xO appears more uniform, with smoother, lower-contrast textures, particularly near the curved meniscus against the CNT wall. Figure R2 provides additional examples of the pixel textures that are typically representative of the amorphous and liquid phases.

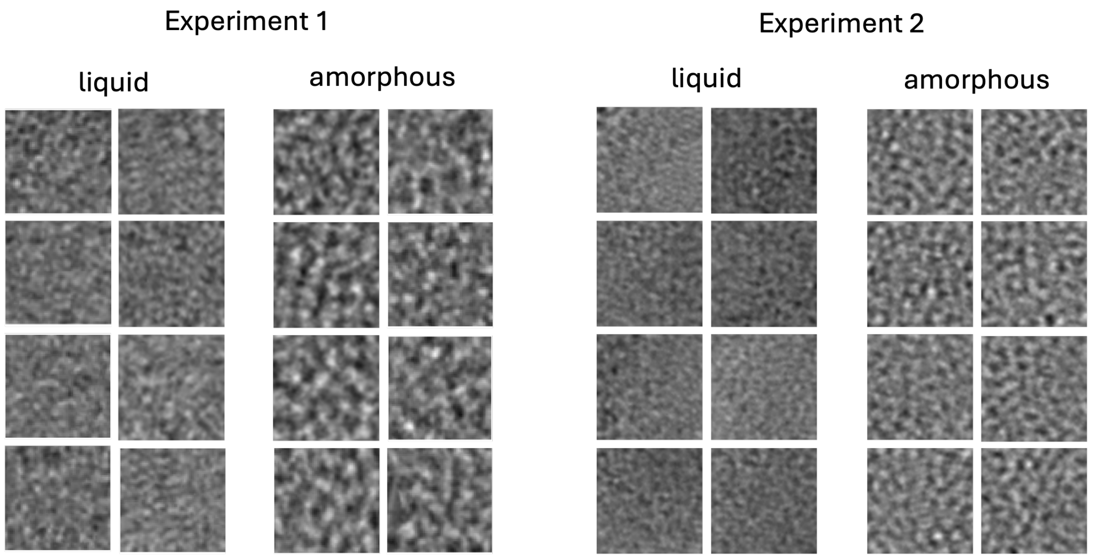

Figure R2: Further examples of 64 x 64 pixel patches micrographs showing these subtle variations contrast and textural distinctions.

The CNN was trained to distinguish between these textures during convolutional operation in initial layers capture edges and gradient patterns, while deeper convolution layers integrate spatial context, such as the position of Sn atoms. The Sn atom show higher contrast in the BF-TEM due to the high electron density and scattering of electrons. In the liquid, Sn atoms are mobile and not station to the electron beam.

These differences are captured by the CNN across the series of convolutional and pooling layers, which are sensitive to spatial frequency and contrast gradients, enabling pixel-wise phase segmentation.

Meniscus as Indicator of Liquid Phase

In both experiments, the presence of a liquid phase was evident at the liquid-vapor interface, where the nanowire contacted the internal wall of the carbon nanotube, particularly at the nanotube core or open end. This interface persisted momentarily as the liquid transitioned into the vapor phase, providing evidence of a dynamic liquid–vapor process.

Figure R3 shows the additional TEM micrographs that were acquired in sequence of ARTEM micrographs showing the retraction of the liquid interface as it enters the vapor phase, for experiment 1 of 2 mins 5 seconds across an average of 29 s.

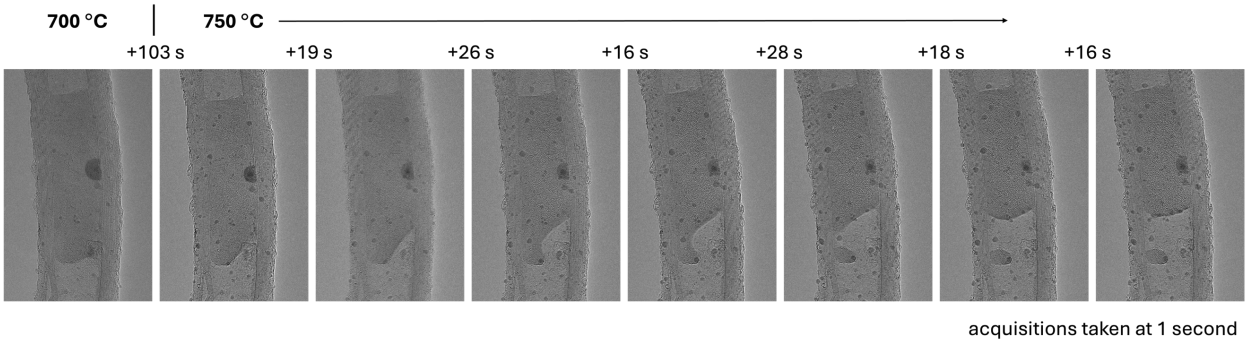

Figure R3: Experiment 1 of the in situ observations of a Sn_xO nanowire as the sample is progress.

Similarly, the same behaviour was also observed for second repeat experiment 2 in Figure R4, which showed a similar retraction of the liquid interface across 3 mins 56 seconds.

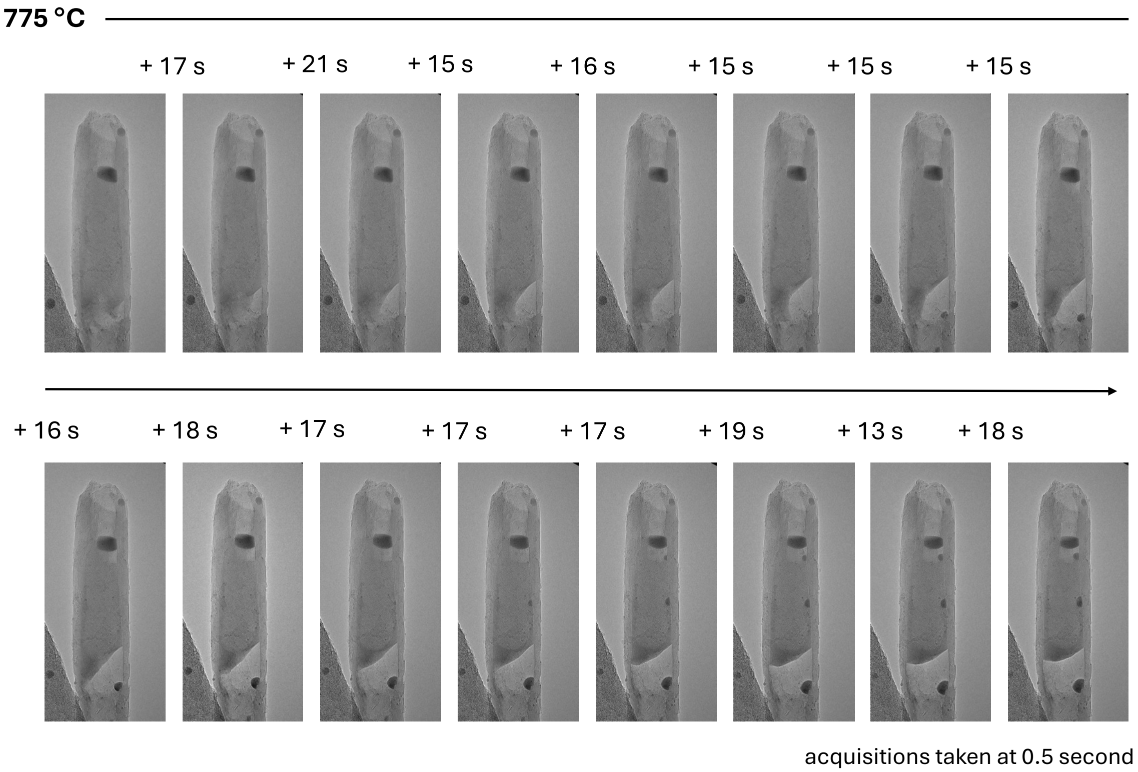

Figure R4: Experiment 2 in situ heating Sn_xO nanowire at 750 ℃, illustrating the Sn_xO melting into the liquid phase and subsequent vaporisation from the retraction of the liquid interface.

Although the microscope was equipped with a Gatan OneView camera capable of high-speed in situ imaging (up to 25 fps, with hardware capability exceeding 1000 fps), we manually selected frame intervals of approximately 15-20 s. This approach was adopted to avoid excessive data generation and was adjusted depending on the dynamic evolution of the sample. A notable delay occurred between temperature ramping and image acquisition, due to thermal distortion of the MEMS chip at elevated temperatures. This distortion shifted the sample out of the focal plane. As a result, manual refocusing within the TEM was necessary before continuing with subsequent imaging sequences.

5. *For the temperature-dependent wetting behaviour of Sn_xO nanowires, what are the speeds of the evolution steps? What is the frame rate of the in-situ imaging, and is it fast enough to capture these transitions?*

Below is an example of the temperature profile used to probe the wetting behaviour of a Sn_xO nanowire to of the DENSsolution heating chip. The experiment was conducted by increasing the temperature in increments and holding the temperature. The temperature was rate slowly and periodically allowing the sample temperature to equilibrate and often leading to dynamic movement in the Sn_xO nanowire, such as for when the temperature was raised for 750 °C.

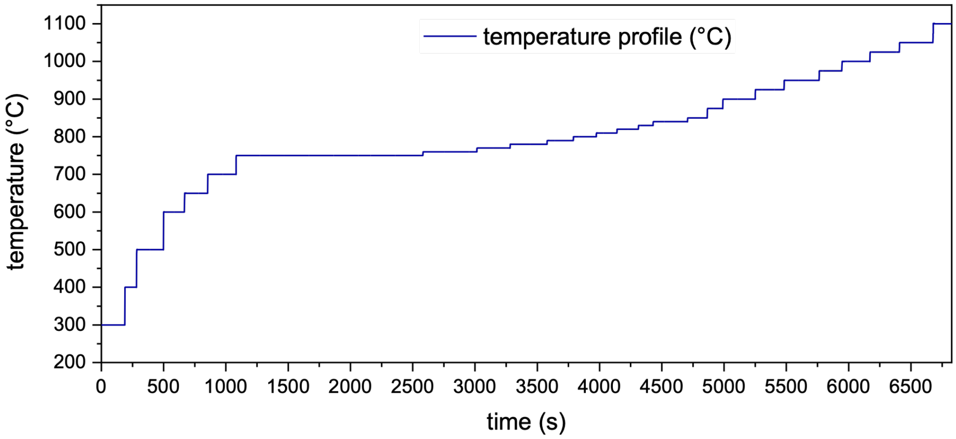

Figure R5: Temperature profile of the experiment 1 DENSsolution heating chip. Applied temperature profile for the DENSsolutions heating chip, showing the ramp and hold sequence, and the duration of the hold used during in situ imaging.

Because of the long duration of the experiment (approximately 2 hrs to full image the process), to prevent excessive data, micrographs were roughly acquired every 20 s which was suitable to capture the dynamic wetting process. For the micrographs of Sn_xO nanowire used for contact angle analysis, 18 mins and 1 second between those micrographs. The graph also provides an indication to the speed of the evolution of the Sn_xO nanowire transformations as they occur. (Specific frame rate for the active retraction of the liquid meniscus and subsequent vaporisation is presented in Figure R3 and R4, respectively above.)

Which transition is most sensitive to incremental temperature changes?

In our system, phase changes were not observed to occur incrementally with temperature. Instead, the most sensitive transition occurs upon reaching the liquid–vapour phase transition temperature, beyond which melting and subsequent vaporisation proceed rapidly. This transition is reflected in the prolonged isothermal hold at 750 °C, during which the liquid phase is maintained before complete vaporisation.

The observation of Sn_3O_4 appearing and disappearing in some figures (e.g., Fig 2, navy blue) requires interpretation.

The observed appearance and disappearance of Sn_3O_4 (navy blue labelled region in Figure 2) can be attributed to two key factors:

Firstly, the disproportionation reaction that leads to the formation of Sn_3O_4 is dynamic and sensitive to the local stoichiometry within the nanowire. Localised changes in oxygen content and redox conditions may cause Sn_3O_4 crystallites to form transiently or dissolve, resulting in intermittent visibility across frames.

Secondly, the visibility of Sn_3O_4 in the CNN segmentation is influenced by the crystallite orientation due to the nature of TEM imaging. Consequently, when a Sn_3O_4 crystallite rotates or slightly realigns during in situ heating, the projected lattice (micrograph contrast pattern) can change substantially. This means that even though the underlying phase remains the same, its appearance in the image may differ. Since the CNN is trained on a finite set of crystal projections, it is more confident in recognising structures that match those trained geometries. If the orientation deviates significantly, the CNN may no longer classify that region as Sn_3O_4, even though the phase is still present. A consideration for future CNN models is improving their ability to account for crystallographic variation and orientation-dependent features.

6. *The CNN training dataset (P4, L123, and P15, L518) involved manually labelling 1,000,000 image patches. This is a very human-intensive task, especially given the quantity and difficulty of distinguishing subtle lattice fringes/patterns.*

Training: Micrograph Labelling

Manual labelling was initially performed on representative ARTEM micrographs, with regions coloured according to distinct structural phases. Since the CNN framework processes the central pixel based on the surrounding 64 × 64 pixel patch texture, training data were generated by extracting patches from these labelled datasets. The model slides this patch window across the image to classify each pixel based on its local context.

Example of training patches extracts from manually labelled ARTEM micrographs are shown in Figure R6.

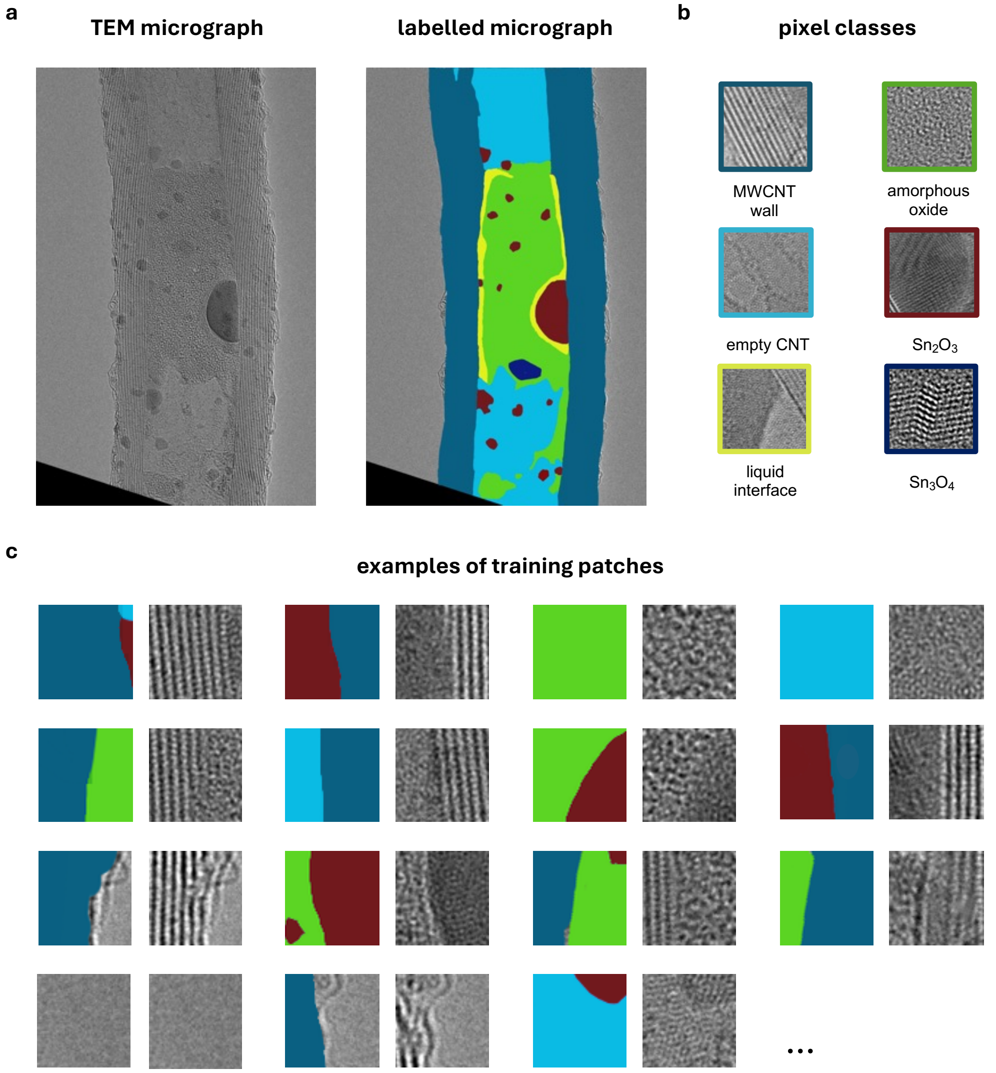

*Figure R6: **a**, TEM micrograph of a Sn_xO particle encapsulated in MWCNT (left), with the corresponding segmented labelled micrograph (right) used to identify the different structural features. **b**, Representative textural patterns used to classify each feature based on the central pixel, illustrating the atomic-scale variations in the micrograph texture learned by the model. Including, the MWCNT wall (blue), amorphous Sn_xO oxide (green), empty core of the MWCNT (light blue), crystalline structure of Sn_2O_3 (dark red) and Sn_3O_4 (dark blue), and the liquid region (yellow) during the solid-liquid transition of the Sn_xO particle. **c**, A selection of 64 × 64 pixel patches, showing both labelled and raw texture examples extracted from multiple TEM micrographs. These patches represent the diverse dataset used to train the CNN, ensuring the model learns to distinguish structural features across different imaging conditions. Examples of these patches, include the boundary between different features, such as the particle and the MWCNT wall, empty core of the tube and the MWCNT wall, and unannotated background because of electrons directly hitting the detector.*

Our CNN learns to recognise each region by looking at many small cut-outs of the micrographs (patches), each labelled according to what material phase the central pixel represents. Each patch contains the local texture and structure of the material phase, which the network learns to associate with a class label (Figure R6b; background, tube wall, oxide, liquid). By seeing thousands of such examples, the CNN gradually learns the characteristic visual patterns of each phase. During application, it then assigns labels to new patches in unseen images, which can be combined to reconstruct a full region map of the micrograph.

Please clarify if any image processing techniques (e.g., FFT filtering, denoising, contrast enhancement) were applied.

CNNs do not require any classical image preprocessing (image enhancement, noise removal, filtering, etc.), therefore we did not apply any. In fact, applying classical preprocessing may be counterproductive, since it may remove texture patterns captured by the convolutional layers. We only scaled pixel intensity values to a consistent range of [0, 1], since this step improves training convergence and stability, and rescaled images from two experiments to a consistent resolution, so that image patch of size 64x64 always covers the same physical area, thus texture patterns are of the same size.

Additionally, what are the classification accuracies for each class? Was there any class imbalance in the training dataset that might have affected detection accuracy?

The model scored a total accuracy of 0.9781 on validation set, and per-class accuracies as follows:

background: 0.9995 (85050 samples)

MWCNT wall:	0.9850 (53295 samples)
empty tube:	0.9450 (25092 samples)
amorphous Sn_xO:	0.9369 (32637 samples)
Sn_xO liquid:	0.6706 (2268 samples)
Sn_3O_4:	0.8393 (336 samples)
Sn_2O_3:	0.7628 (5022 samples)

The dataset was imbalanced, which reflects the frequency of different regions in ARTEM micrographs (e.g., background is dominant, whereas Sn oxide or liquid occurs only locally). While this can lower scores for minority classes, the effect on region analysis is limited: dominant regions are quantified very accurately, and the less represented classes are still detected reliably, though their areas may be slightly underestimated due to both imbalance and imperfections in manual annotations. Since our goal was to use the model as a labelling tool rather than to optimise classification performance, the obtained accuracies are sufficient to support a quantitative assessment of regions in the micrographs.

It is suggested to provide more details on the challenges and limitations of the developed model, considering the characteristics of these datasets.

Model training: accounting for variation in the dataset

ARTEM Modification Considerations for Deep Learning Analysis

The accuracy of CNNs for classifying atomic-scale features from ARTEM data is highly dependent on the quality, resolution, and consistency of input micrographs. Several imaging parameters (particularly magnification, spatial sampling, and focal depth) provide variations in contrast and feature representation. These experimental factors must be accounted for to ensure robust and generalisable model performance.

Impact of Magnification and Pixel Sampling

In ARTEM, magnification directly affects the projection of atomic structures onto the detector, in this case, a Gatan OneView CMOS camera with a 4096 × 4096 pixel array. At higher magnifications, atomic features such as lattice fringes and graphitic layers are projected over more pixels, enhancing spatial resolution. At lower magnifications, the same features are compressed, leading to fewer pixels per atom and loss of critical detail. This variation in pixel density alters how convolutional filters interpret and extract features, potentially limiting classification fidelity.

To address this, all micrographs were spatially normalized to a consistent pixel representation of atomic features. Specifically, the number of pixels per carbon atom in the MWCNT wall was equalised across datasets using bicubic interpolation, which provides smooth scaling by calculating new pixel intensities based on a weighted average of the 16 nearest neighbours. Both upsampling and downsampling were applied, depending on the original magnification, ensuring consistent spatial frequency and contrast patterns for CNN training.

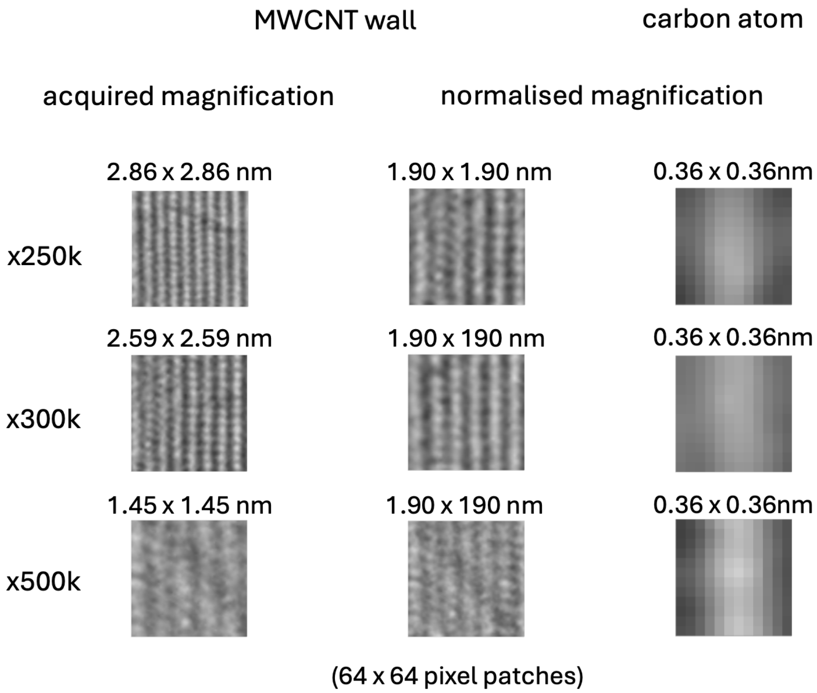

Figure R7: illustrates how pixel density varies with magnification and how interpolation standardises representation. This normalization enables CNNs to extract comparable low-level and high-level features regardless of the original imaging scale.

Bicubic interpolation is well-suited for resizing electron microscopy images, where contrast is governed by electron scattering, as it preserves local intensity gradients and smooth transitions between features. This helps maintain the fidelity of scattering-based contrast, which is essential for accurate structural interpretation.

Focus Plane Variation and Influence on Contrast

Another critical factor is the focal depth of the microscope during imaging. At 80 kV acceleration voltage under near-parallel illumination conditions, the JEOL ARM300CF provides a large nominal depth of field on the order of several microns. However, due to

the limited coherence envelope and defocus spread, only a narrow axial slice, 2–3 nm—contributes to sharply resolved lattice contrast at any given focal plane. Given the cylindrical geometry of encapsulated nanostructures, such as those confined within carbon nanotubes, features located away from this thin imaging plane appear blurred or exhibit reduced contrast. As a result, apparent intensity differences in HRTEM images can arise from slight axial displacements, even if the projected mass remains constant.

This depth sensitivity is particularly relevant when interpreting crystalline versus amorphous textures. For instance, a crystalline Sn_2O_3 nanoparticle will exhibit distinct lattice fringes and diffraction contrast when aligned with the focal plane. However, even slight axial displacement can suppress these features, making the particle appear amorphous. Conversely, graphitic MWCNT walls may exhibit intensified interference fringes under overfocus due to enhanced phase contrast.

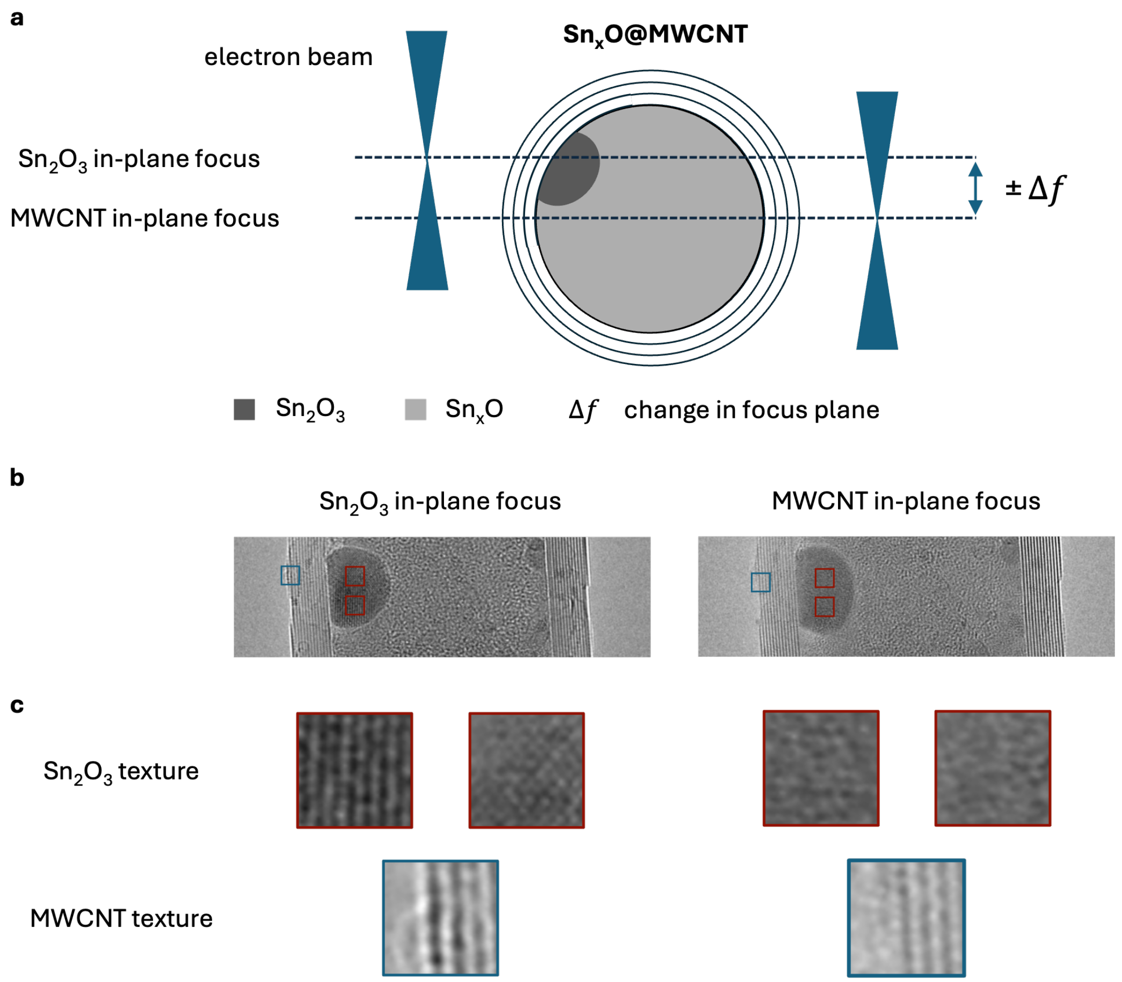

Figure R8: Comparative ARTEM micrographs and a schematic showing how focal variation affects contrast. The appearance and apparent crystalline or amorphous phase of encapsulated particles can visually change depending on their relative position to the focal plane.

Rotation of crystallites

One of the key limitations encountered during CNN-based segmentation was the challenge of recognising crystallites when viewed along projections do not present in the training dataset. Crystals, particularly intermediate oxides such as Sn_2O_3 or Sn_3O_4, can exhibit varying contrast patterns depending on their orientation relative to the electron beam. Unless these specific orientations are explicitly included during training, the CNN model may fail to correctly classify the corresponding atomic pattern, instead labelling it as ambiguous or non-crystalline. This sensitivity to crystallographic projection highlights the need for either training on a broader diversity of simulated or experimentally annotated projections or incorporating rotationally invariant model architectures.

7. *In SI, Fig. S5 (r-s), were there mixed phases that occurred, as evidenced by the formation of regions in purple?*

Fig. S5 (r-2) have been updated accordingly. A labelling colour error.

Did you observe any beam damage or electron-dose sensitivity behaviour for some phases/components?

Electron beam damage to the Sn_xO@MWCNT system is unavoidable, even when imaging at reduced accelerating voltages. ARTEM imaging was therefore conducted at 80 kV to minimise such damage, which primarily arises from direct momentum transfer between high-energy electrons and atomic nuclei. These knock-on events transfer kinetic energy into the lattice, which is rapidly converted into heat, leading to a local and artificial rise in sample temperature. At higher accelerating voltages, the severity of this effect increases, often resulting in thermal artefacts such as beam-induced melting that obscure genuine temperature-driven transformations. By operating at 80 kV, we ensured that the dynamic behaviours observed (such as melting, wetting, and vaporisation) were predominantly governed by controlled thermal input rather than beam-induced effects.

Minor suggestions:

***1.** P2, L68 – remove "recent"; else if there is additional text after, please add the same.*

Amended.

***2.** P2, L85 – "Analysis of both the cubic and tetragonal phases of β-Sn is shown in Extended Data Figures." Are the authors referring to the cubic β-Sn metastable phase and the tetragonal β-Sn stable phase here?*

Revised (P2, L141-144) : Extended Data Figures 1 and 2 show β-Sn imaged along two orthogonal zone axes – one projection appears pseudo-cubic crystal face due to its viewing direction, while the other clearly reveals the characteristic tetragonal symmetry of the β-Sn structure.

***3.** Several figure numbering issues. P6, L152, "Figure 2d-u" should be "Figure 2b-u", L159, "Figure 2d" should be "Figure 2b", L162, "Figure S4c-q" should be "Extended Data Figure 2". P20 L689, "figure 2 d-w" should be "figure 2 b-u". All figures in the SI, section 3.*

Thank you for catching these. Figure number has been checked and updated.

***4.** P9, L275, replace "time" with "vapor annealing time" for clarification, as these are not in-situ real-time observations, but rather static observations of samples prepared using different vapor annealing times.*

Amended.

***5.** In the SI, section 6 EELS analysis, P18, Figure S9's caption, "Showing the low-loss spectrum for the carbon K-edge(284 eV) and the high-loss spectrum for the oxygen K-edge(532 eV)", remove" low-loss spectrum" and "high-loss spectrum" as both C-K and O-K edges are core-loss features. Figure S14 for Sn M4,5 edges was missing.*

Please find the updates figure, caption, and associates text in the tracked manuscript. P19; L 535-651.

***6.** In the SI, Fig. S7 is missing scale bar information.*

Updated. P18, L515; Scale bar: **a**, 2 nm, **b**, 0.7 nm, **c**, 5 nm^{-1} **d**, 0.7 nm.

***7.** In the SI, P8 – "ARTEM micrographs exhibit significant contrast variance, as the micrograph intensity depends on the interference of electron waves that pass through and exit the sample. This phase contrast is highly sensitive to experimental conditions such as defocus, sample thickness, and beam brightness, leading to variations in the appearance of atomic features. This contrast is highly sensitive to experimental TEM parameters,*

including defocus, beam brightness, and sample thickness, all of which can alter the appearance of atomic-scale features (either as bright or dark contrast)."
The last two sentences are repeated.

Revised.

8. *In the SI, P18 – "The localized distribution of the oxygen-K edge signal, contrasts with the continuous wetting layer that would be expected if classical vapour condensation mechanisms." It seems that there is some text missing at the end of this sentence.*

Added P19; L552-556: The spatially localised O-K edge signal is confined to the Sn_xO nanowire and absent from the inner nanotube wall. This stands in contrast to classical condensation models, which predict the formation of continuous thin wetting films via adsorbed liquid layers on confining surfaces prior to condensation. The absence of such a signal suggests that pre-wetting layers do not form under the extreme confinement of the MWCNT core.

9. *In the SI, P20 – "The LW model describes the capillary rise of a liquid into a cylindrical core of the tube, balancing the driving capillary pressure against, which drives the liquid flow, against the viscous drag, opposed the advancing meniscus."*
Against (the first instance in the above sentence should be removed). Additionally, should "opposed" be changed to "which opposes"?

Added P21, L694-696. The LW model describes the capillary rise of a liquid into a cylindrical core of the tube, balancing the driving capillary pressure, which drives the liquid flow, against the viscous drag, which opposes the advancing meniscus.

10. *In the SI, P21 – Figure S11 The power law prefactor in Figure S11 (b) should be 0.92 instead of 0.0.92 Figure S11(c) and (b) have the same caption. Should (c) be 12 hr annealing time? If yes, please modify the caption on Page 22.*

Corrected.

11. *In the SI, P23 – Figure S12 and Table S7 The fit parameters for Non-Fixed Power-Law Model in Figure S12 (a)-(d) and Table S7 do not match. If both prefactor and power law scaling exponent were floated, how likely is it that the pre-factors in Table S7 and S6 match? Please check.*

Graphs and associated fitting parameters are updated accordingly in SI.

12. *Can the authors discuss the effects of MWCNT surface chemistry/defects on metal nanowire wetting? Additionally, experimental techniques such as in-situ 4D STEM and atomic-scale simulations (DFT, AIMD, etc) could further elucidate the fundamental*

mechanism of this phenomenon. Expanding the study to include these approaches in the future would be beneficial.

Although, as we have presented, characterising and understanding the wetting dynamics inside MWCNTs is challenging, particularly at the nanoscale environment, the variation in the contact angle within this study indicate that the wetting of metal nanowires on MWCNTs is influenced by both surface chemistry and structural defects. Given that the internal structure of an MWCNT resembles cylindrical graphene, insights from graphene studies indicate the influence of atomic defects. For example, molecular dynamics simulations by Li et al.[1] demonstrated that introducing even a small density of monoatomic vacancies makes graphene less wettable by water. Their simulations showed that the contact angle of water on pristine graphene was 92 °, which increased to 99 ° upon introducing 2% monoatomic vacancies. These vacancy sites reduce solid-water interactions, such as fewer favourable contact points and disruption of the π-electron surface and increasing the contact angle and lowering the effective surface energy. Similarly, simulations on graphene oxide showed that the introduction of holes or vacancies decreases hydrophilicity: the water contact angle rose from 70 ° (no vacancies) to 82 ° when 10% of carbon atoms were removed to introduce atomic defects[2].

Further investigation to corroborate 4D-STEM for defect analysis and in situ wetting dynamics could be used to help understand within these extreme environmental conditions (high vacuum and high temperature).

References

1. Li, X., Li, L., Wang, Y., Li, H. & Bian, X. Wetting and interfacial properties of water on the defective graphene. *Journal of Physical Chemistry C* **117**, 14106–14112 (2013).
2. Kozbial, A. *et al*. Study on the surface energy of graphene by contact angle measurements. *Langmuir* **30**, 8598–8606 (2014).

Reviewer #3 (Remarks to the Author):

I co-reviewed this manuscript with one of the reviewers who provided the listed reports. This is part of the Nature Communications initiative to facilitate training in peer review and to provide appropriate recognition for Early Career Researchers who co-review manuscripts.
